# Synthetic metabolic computation in a bioluminescence-sensing system

**DOI:** 10.1093/nar/gkz807

**Published:** 2019-09-23

**Authors:** Natalia Barger, Phyana Litovco, Ximing Li, Mouna Habib, Ramez Daniel

**Affiliations:** Department of Biomedical Engineering, Technion - Israel Institute of Technology, Haifa 3200003, Israel

## Abstract

Bioluminescence is visible light produced and emitted by living cells using various biological systems (e.g. *luxCDABE* cassette). Today, this phenomenon is widely exploited in biological research, biotechnology and medical applications as a quantitative technique for the detection of biological signals. However, this technique has mostly been used to detect a single input only. In this work, we re-engineered the complex genetic structure of *luxCDABE* cassette to build a biological unit that can detect multi-inputs, process the cellular information and report the computation results. We first split the *luxCDABE* operon into several parts to create a genetic circuit that can compute a soft minimum in living cells. Then, we used the new design to implement an AND logic function with better performance as compared to AND logic functions based on protein-protein interactions. Furthermore, by controlling the reverse reaction of the *luxCDABE* cassette independently from the forward reaction, we built a comparator with a programmable detection threshold. Finally, we applied the redesigned cassette to build an incoherent feedforward loop that reduced the unwanted crosstalk between stress-responsive promoters (*recA, katG*). This work demonstrates the construction of genetic circuits that combine regulations of gene expression with metabolic pathways, for sensing and computing in living cells.

## INTRODUCTION

Over the past few decades, bioluminescence-sensing systems, such as those in the firefly and bacterial luciferase, have gained widespread attention as a low-cost tool for live organism imaging and biological signal detection (1–3). Such systems have been utilized to detect and monitor environmentally toxic chemicals in air, water and food (4,5). Recently, it has been shown that bioluminescence-sensing systems can also monitor the gastro intestine, wirelessly communicating information to an external computer (6). Another application is studying the dynamics of gene expression at a single cell level (7). Bioluminescence-sensing systems are genetically engineered cells that can recognize target molecules and convert biological responses to light, which can then be measured by a physical detector (8). Since biological systems contain many highly evolved biochemical pathways, biosensors that are based on living cells are sensitive to a much wider range of chemicals and analytes than chemical biosensors. Accordingly, here, we exploited the complex genetic structure and biochemical pathway of the bacterial bioluminescence, using the *luxCDABE* cassette (9), and redesigned it to build several bacterial biosensors, that can detect and integrate multiple signals with computing and decision-making capabilities. Our design enables scaling the computational complexity of synthetic gene and molecular networks, using minimal components in the context of synthetic biology.

Natural biological systems employ transcriptional and translational regulation of gene expression and metabolic pathways to process biological and environmental signals for decision making and actuation. In contrast, synthetic gene circuits often use transcription and translation regulation to control gene expression but without utilizing enzymatic reactions as part of the computation. In such cases, the transfer function of the synthetic gene circuit is determined only by the promoter activity. For example, transcriptional and translational elements have been used to build artificial logic gates (10–13), counters (14), memory (15–17) and analog circuits (18) in living cells. However, the often unwanted interactions between synthetic parts and host cells result in toxicity (19) and over consumption of cellular resources (20,21), poses a challenge in scaling the complexity of synthetic gene networks to the required computational level. Metabolic engineering for computation has rarely been demonstrated in the framework of synthetic biology (22–24), whereby a transfer function of synthetic metabolic circuit is set by the interaction between the forward and reverse enzymatic reactions. The ability to control both directions of enzymatic reactions, can give rise to the development of complex computational networks by using fewer synthetic parts compared to conventional networks (e.g. circuits that are controlled by transcription factors only). For example, it has been shown that controlling the phosphatase level of the two-component regulatory system can tune the detection threshold of the system (25), where the detection threshold is defined as the input concentration when the output state is switched between low and high.

The bioluminescent *luxCDABE* cassette, which underlies the visible light emission in bacteria (e.g*. Aliivibrio fischeri* or *Photorhabdus luminescens*), includes five genes that can enhance or inhibit light emission with a peak at 490 nm (26). The *luxAB* genes encode luciferase, which is the catalyst of the reaction. The bacterial luminescence reaction involves the oxidation of a long-chain aliphatic aldehyde$\ ( {R - CHO} )$, and flavin mononucleotide $(FMN{H_2})$ resulting in the release of excess free energy in the form of light. The *luxCDE* genes encode for three enzymes (reductase, transferase and synthetase) that are needed to produce substrate for the luminescence reaction (aldehyde). The enzyme transferase reacts with acyl-ACP from the biosynthesis pathway and releases free fatty acids $( {R - COOH} )$, which are then reduced to an aldehyde by synthetase and reductase.

In this work, we built an artificial engineering framework for bacterial biosensors that combines the benefits of transcriptional gene expression regulatory elements and the metabolic *luxCDABE* cassette pathway. We first redesigned the *luxCDABE* cassette by splitting the five genes into different combinations. Then, we integrated synthetic inducible and environmental stress promoters, such as *recA* (27) and *katG* (28), into the engineered cassette to improve the detection specificity of bacterial biosensors. The proposed system has several advantages over other synthetic bio-sensing systems. It can be used for imaging and also for complex computations such as analog (18), digital (29) and mixed-signal processing (30), with fewer synthetic components and parts. Furthermore, the expression of all *lux* operon genes within living cells allows for a fully independent light generation system requiring no additional substrates or excitation by an external light source (e.g. a green fluorescent protein) (2). Moreover, in contrast to previously developed metabolic circuits in living cells, that often require fusion of a reporter protein (31) or protein assay (24) to characterize the activity of the metabolic circuits, the *luxCDABE* cassette activity can be measured directly.

## MATERIALS AND METHODS

### Chemicals

All chemicals were of the highest analytical grade. Nalidixic acid (NA) and hydrogen peroxide (H_2_O_2_) were obtained from Sigma-Aldrich. Arabinose and acyl homoserine lactone 3OC6HSL (AHL) (Sigma-Aldrich) were used as inducers.

### Bacterial strains and gene origins

The *Escherichia coli* (*E. coli*) 10β strain (see Section 1, Supplementary Table S1 of the Supplementary Data for genotype) was used to construct all plasmids and the *E. coli* MG1655 wild type strain (see Section 1, Supplementary Table S1 of the Supplementary Data for genotype) was used for stress-responsive promoter kinetics and activity assays.

Plasmid pBR-2TTS-pLux, which harbors the *Photorhabdus luminescens luxCDABE* genes (GenBank accession number M90093) downstream of a multiple cloning site, and pACYC-*luxCDE*, were kindly supplied by the S. Belkin lab and served as the source of the *P. luminescens lux* genes. Each individual gene (*luxA, luxB, luxC, luxD, luxE*) and each functional unit (*luxAB* and *luxCDE*) were isolated by PCR amplification, using primers that introduced an Acc65I/KpnI restriction site before each gene/unit and a BamHI/XmaI restriction site after each gene/unit (Section 1, Supplementary Table S2 of the Supplementary Data). The *E. coli* strain MG1655 chromosomal DNA was used as a template for the PCR amplification of the *katG* and *recA* gene promoters (see Section 1, Supplementary Table S2 of the Supplementary Data for primers).

### Plasmid construction

All the plasmids in this work (see Section 1, Supplementary Figure S1 and Supplementary Table S3 of the Supplementary Data for plasmid maps and combinations) were constructed using basic molecular cloning techniques (32). Modifications of plasmids were confirmed by restriction digests, performed using New England Biolabs (Beverly, MA) restriction endonucleases and Thermo Scientific FastDigest Restriction Enzymes. For ligation, T4 DNA ligase, and Taq polymerase were used. PCR was performed with a Bio-Rad S1000™ Thermal Cycler with Dual 48/48 Fast Reaction Modules. Synthetic oligonucleotides were synthesized by Integrated DNA Technologies (Coralville, IA). Manipulation of different parts of the same element (promoters or genes) (see Section 8, Supplementary Table S11 the Supplementary Data for genetic parts) was carried out using the same restriction sites. To assemble three or more parts, we used the Gibson Assembly Master Mix from New England Biolabs (Ipswich, MA), as per the manufacturer's instructions. Plasmids were transformed into *E. coli* using a standard heat shock protocol (32) or a MicroPulser electroporator (33). Colony PCR screening was carried out using forward and reverse primer pairs. Positive clones were sequence-verified. Plasmids were isolated with Qiagen QIAprep Spin Miniprep Kits (Qiagen, Hilden, Germany), according to the manufacturer's instructions. DNA sequencing was carried out by the Macrogen Sequencing Service (Macrogen Europe, The Netherlands).

### Circuit characterization

*E. coli* strains were grown from glycerol freezer stocks at 37°C, in a Shel Labs SSI5 shaking incubator at 250 rpm, in 5 ml Luria-Bertani-Miller (LB) medium (Fisher), supplemented with carbenicillin (50 μg m^−1^), kanamycin (30 μg m^−1^) or chloramphenicol (25 μg m^−1^). Overnight cultures were diluted 100-fold into 5 ml fresh LB medium with antibiotics and regrown with shaking at 37°C and 250 rpm to the early exponential growth phase (OD_600_ ≈ 0.12). Culture aliquots (200 μl) were then transferred into the wells of 96-well plates, containing inducers or test chemicals diluted in LB or a toxicant-free control (LB only). Following a 4 hours incubation at 37°C, luminescence and green fluorescent protein (GFP) signals were read at 15 min intervals using a Synergy H1 monochromator-based multi-mode microplate reader with a microplate shaker (500 rpm). The luminescence signal was measured at 490 nm and GFP fluorescence was quantified by excitation at a wavelength of 488 nm and emission at a wavelength of 510 nm. All experiments were repeated three times. Luminescence and GFP values were measured in arbitrary relative luminescence units (a.u.) and normalized by the optical density of the culture measured at 600 nm.

### Designing and modeling of a bioluminescent *luxCDABE* cassette

**Figure 1.**
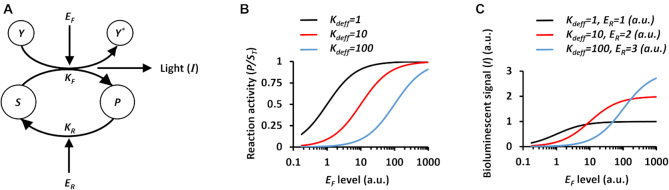
Bacterial bioluminescence model. (**A**) Scheme of light generation by *luxCDABE* cassette, where $S$ represents the concentration of $R - CHO$, $P$ represents the concentration of $R - COOH$, $Y$ represents the concentration of $FMN{H_2}$ and ${Y^*}$ represents the concentration of $FMN$. ${E_{F\ }}$ represents the expression level of the enzyme involved in the forward reaction (luciferase) and ${E_{R\ }}$ represents the expression level of enzymes involved in the reverse reaction (transferase, synthetase, and reductase). ${K_F}$ represents the rate of the forward reaction and ${K_R}$ represents the rate of the reverse reaction. (B) Simulation results demonstrating the relationship between the ${E_{F\ }}$ level and the reaction activity at varying ${E_{R\ }}$ levels, where $P$ is normalized by ${S_T}$, the total concentration of the $S$ molecule. ${K_{deff}}$ can be viewed as a detection threshold that depends on the level of ${E_{R\ }}$ according to $\ {K_{deff}} \equiv \frac{{{K_R} \cdot {E_R}}}{{{K_F}}}$. (**C**) Simulation results demonstrating the relationship between the ${E_{F\ }}$level and bioluminescent signal (*I*), at varying ${E_{R\ }}$ levels.

The process of light generation (bioluminescent signal) by the *luxCDABE* cassette, is given by the following two reactions (26):

(1) $$\begin{eqnarray*}FMN{H_2} &+& R - CHO + {O_2}\mathop{-\!\!\!-\!\!\!-\!\!\!-\!\!\!\longrightarrow}^{{\rm{luciferase}}} FMN \nonumber\\ &+& R - COOH + {H_2}O + Light\end{eqnarray*}$$

(2) $$\begin{eqnarray*}R - COOH &+& ATP + NADPH\mathop {-\!\!\!-\!\!\!-\!\!\!-\!\!\!-\!\!\!-\!\!\!\longrightarrow}^{\begin{array}{c} \scriptstyle{\rm reductase}\\ \scriptstyle{\rm synthetase} \end{array}} R - CHO \nonumber\\ &+& AMP + PP + NAD{P^ + },\end{eqnarray*}$$

where $R - CHO$ is a long-chain aliphatic aldehyde, $FMN{H_2}$ is a reduced flavin mononucleotide, ${O_2}$ is oxygen, ${H_2}O$ is water, $FMN$ is flavin mononucleotide and $R - COOH$ is the fatty acid. $ATP$ is adenosine triphosphate and $AMP$ is adenosine monophosphate, both of which are molecules that act as energy carriers. $NADPH$ and $NAD{P^ + }$ are nicotinamide adenine dinucleotide phosphate molecules in reduced and oxidized forms, respectively, which facilitate electron transfer, and $PP$ is diphosphate (see Section 2, Supplementary Table S4 of the Supplementary Data for list of parameters). A simple model (Figure 1A) that describes reactions 1 and 2 is given by:

(3) $$\begin{equation*}\ \frac{{dP}}{{dt}} = {K_F} \cdot {E_F} \cdot S - {K_R} \cdot {E_R} \cdot P\end{equation*}$$

(4) $$\begin{equation*}\frac{{dP}}{{dt}} = - \frac{{dS}}{{dt}},\end{equation*}$$

where ${K_F}$ is the rate of the forward reaction which is catalyzed by luciferase $({E_{F}})$, and ${K_R}$ is the rate of the reverse reaction which is catalyzed by reductase and synthetase $({E_{R}})$. The variables $S$ and $P$ represent the concentrations of $R - CHO$ and $R - COOH$, respectively. $Y$ and ${Y^*}$ (in Figure 1A) represent the concentrations of $FMN{H_2}$ and $FMN$, respectively. At the steady state, the concentration of $P$ is equal to:

(5) $$\begin{equation*}P\ = {S_T}\ \cdot \frac{{\frac{{{E_F}}}{{{K_{deff}}}}}}{{1 + \frac{{{E_F}}}{{{K_{deff}}}}}},\end{equation*}$$

where ${S_T}$ is the total concentration of the $S$ molecule, and ${K_{deff}} \equiv \frac{{{K_R} \cdot {E_R}}}{{{K_F}}}$. The model (Equation 5) indicates that on the one hand, enhancing the expression level of the enzyme ${E_F}$ involved in the forward reaction, increases the level of the product $P$, which is in agreement with the Michaelis-Menten model at steady state (34). On the other hand, increasing the expression level of the enzyme ${E_R}$ involved in the reverse reaction, raises the detection threshold (Figure 1B). Thus, the parameter ${K_{deff}}$ can be viewed as a detection threshold that depends on the level of ${E_R}$. Interestingly, we also found that the kinetics of substances that are involved in the metabolic pathways (e.g. $FMN$ in the *luxCDABE* cassette pathway) can provide extra information beyond the threshold shifting. Continuing with our model (Figure 1A), we assumed that the bioluminescent signal ($I$*)* is proportional to the kinetics of $FMN$ (${Y^*}$), $I \propto {\raise0.7ex\hbox{${d{Y^*}}$} \!\mathord{/ {\vphantom {{d{Y^*}} {dt}}}} \!\lower0.7ex\hbox{${dt}$}}$, where the kinetics of ${Y^*}$ is determined by the forward reaction ($S \to P)$ and the initial concentration of $FMN{H_2}$$( {{Y_{T}}} )$. In cases where the change in the initial concentration of $FMN$ is very small $({Y_{T}} \gg {{Y}^*})$ we can approximate the bioluminescent signal (*I*) as:

(6) $$\begin{equation*}I \propto \frac{{dY^*}}{{dt}} = \frac{{{Y_T}}}{{{K_d}}} \cdot {K_F} \cdot {E_F} \cdot S,\end{equation*}$$

where the ${K_d}$ is the dissociation constant of binding $Y$ to ${E_R}$. By substituting Equation (5) into Equation (6) and using the fact that $S\ = {S_T}\ - P$, the bioluminescent signal is given by:

(7) $$\begin{equation*}I \propto \frac{{{S_T} \cdot {Y_T} \cdot {K_R}}}{{{K_d}}} \cdot {E_R} \cdot \frac{{\frac{{{E_F}}}{{{K_{deff}}}}}}{{1 + \frac{{{E_F}}}{{{K_{deff}}}}}}\end{equation*}$$

The bioluminescent signal model (Equation 7) indicates that enhancing the expression level of *E_R_* not only increases the detection threshold but also increases the signal intensity (Figure 1C). Such a characteristic can be very useful when building complex functions in living cells, because the saturation level (i.e. the maximum level achieved by the reaction when input is very high), and the detection threshold can both be programmed. Notably, this design capability cannot be achieved in circuits based on Equation (5), where the detection threshold is the only parameter that can be controlled (25), or circuit based promoters, where the saturation level is the only parameter that can be controlled (10). Recently advanced genetic circuits that use transcriptional interference containing several programmable design parameters, were successfully constructed (35). However, such circuits required the implementation of complex constructs and large numbers of synthetic parts.

## RESULTS

**Figure 2.**
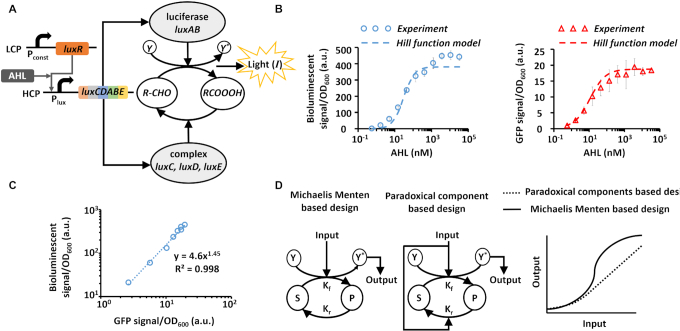
Studying the *luxCDABE* operon characteristics. (**A**) The *luxCDABE* operon is regulated by the P_lux_ promoter located on a high-copy-number plasmid (HCP). LuxR is regulated by a constitutive promoter located on a low-copy-number plasmid (LCP) and is induced by acyl-homoserine-lactone (AHL). (**B**) Measured transfer function between AHL input concentration and either a green fluorescent protein (GFP, right) or bioluminescent (left) outputs. The dashed lines are fitted to Hill functions (for GFP according to $\frac{{AHL}}{{AHL + 11}}$ and for bioluminescence according to $\frac{{AH{L^{1.5}}}}{{AH{L^{1.5}} + 30}}$). (**C**) The bioluminescent signal and the GFP signal comparison, relative to the same levels of AHL concentration. The GFP level regulated by P_lux_ promoter are proportional to *lux* enzymes (**D**) Schematic model showing the linear behaviour of a system with paradoxical components compared to the non-linear behaviour of the Michaelis-Menten model. The average and errors (s.e.m.) shown in the figures are derived from three experiments.

To study the behaviour of the *luxCDABE* cassette which appears often as an operon in natural and synthetic systems, we first constructed a circuit that regulates the *luxCDABE* cassette with an inducible P_lux_ promoter using the quorum-sensing transcriptional activator LuxR (Figure 2A). LuxR was expressed under a constitutive promoter, located on a low-copy-number plasmid (LCP), and induced by acyl homoserine lactone (AHL). The AHL-LuxR complex activates the promoter P_lux_ (Figure 2A), which regulates both luciferase (a heterodimer of *luxA* and *luxB*) and the enzymes required for the production of its substrate aliphatic aldehyde (*luxC, luxD*, and *luxE*). The *luxCDABE* operon was located on a high-copy-number plasmid (HCP). The circuit activity, which is measured by the transfer function between AHL and the bioluminescent signal, is determined by a cascade of P_lux_ activity and the metabolic reactions of the *luxCDABE* cassette (Figure 2B). Then, the *luxCDABE* operon was replaced by GFP, whose expression level is directly proportional to the activity of P_lux_ promoter. The measured transfer functions of AHL-GFP and AHL-bioluminescence are well-matched by Hill-functions (Figure 2B).

Since the five genes of the *luxCDABE* operon are regulated by the same P_lux_ promoter, we can assume that the expression levels of these genes are proportional to the same input ($x$). As we mentioned above, luciferase is formed by the binding of LuxA and LuxB (${E_F} = \frac{{LuxA \cdot LuxB}}{{{K_{AB}}}}$, where ${K_{AB}}$ is dissociation constant), and therefore, we can approximate the level of luciferase concentration as a square function of input $x\ ( {{E_F} \propto {x^2}} )$. The reductase, transferase and synthetase, are produced from the *luxC, luxD*, and *luxE* genes independently, and therefore their concentrations can be approximated as a linear function with input $x$ (${E_R} \propto x$). Thus, and according to Equation (7), the bioluminescence signal can also be approximated by a power-law function with the input $x$ ($I \propto {x^n})$, where $1 < n < 2$. To gain deeper insight into the biophysical model of the wild type circuit, we compared the bioluminescent signal and the GFP signal relative to the same levels of AHL concentration (Figure 2C). The resulting transfer function, that describes the behavior of the *luxCDABE* operon without the contribution of the P_lux_/LuxR system, is fitted by a power-law relation ($I \propto GF{P^{1.45}}$). Based on our analysis, the bioluminescent signal of the wild type circuit is ‘tailored’ to respond in a dose-dependent manner to changes in the input concentrations with an amplification factor (gain) that cannot be achieved by fluorescent protein reporters. Furthermore, the design principle of the wild type *luxCDABE* cassette, based on the regulation of paradoxical components with the same signal (36) (e.g. increasing both the forward and reverse reaction rates of the same metabolic pathway, Figure 2D), allows to convert non-linear relations (e.g. Michaelis–Menten model or Hill-function, Figure 2D) to linear or power-law relations, where saturation is reached for very high input levels only.

**Figure 3.**
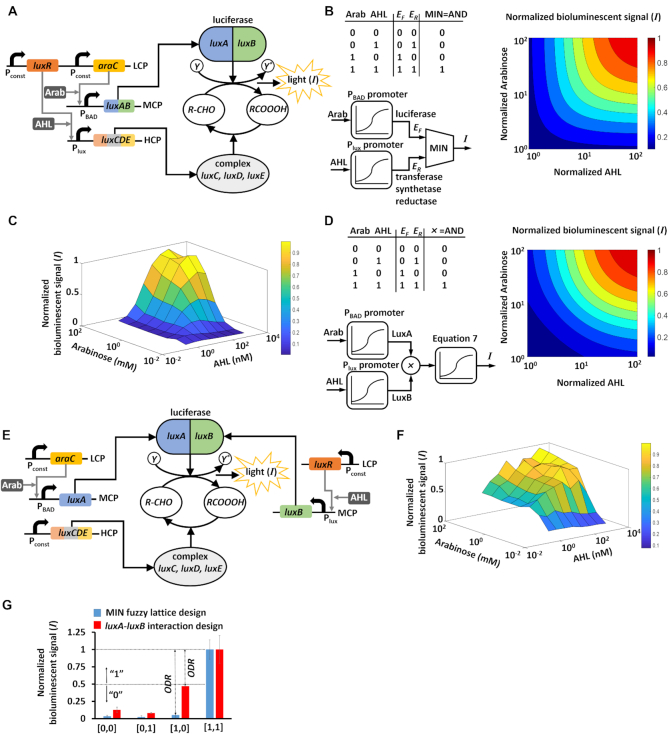
A synthetic MIN fuzzy lattice (soft minimum function) in living cells implemented by splitting the *luxCDABE* cassette. (**A**) *The luxCDE-luxAB* spitting design. The *luxCDE* is regulated by the P_lux_ promoter located on a high copy number plasmid (HCP). LuxR is regulated by a constitutive promoter located on a low copy number plasmid (LCP) and is induced by acyl-homoserine-lactone (AHL) to activate P_lux_. The *luxAB* is regulated by the P_BAD_ promoter located on a medium copy number plasmid (MCP). AraC is regulated by a constitutive promoter located on a LCP and is induced by Arabinose (Arab) to activate P_BAD_. (**B**) Model simulations of the synthetic MIN fuzzy lattice. The model consists of an Arab-${E_F}$ transfer function ${E_F} = \frac{{Arab/{K_{Arab}}}}{{1 + Arab/{K_{Arab}}}}$, an AHL-${E_R}$ transfer function ${E_R} = \frac{{AHL/{K_{AHL}}}}{{1 + AHL/{K_{AHL}}}}$, and a minimum function $Out\ = \frac{{{E_R} \cdot {E_F}}}{{{E_R} + {E_F}}}$, where ${K_{Arab}}$ and ${K_{AHL}}$ have units of concentration and are proportional to the dissociation constants of binding Arab-AraC and P_BAD_, AHL-LuxR and P_lux_. (**C**) The measured AHL/Arabinose transfer function and bioluminescence output for the MIN fuzzy lattice. (**D**) Model of the synthetic AND logic gate based on LuxA-LuxB interaction. The model consists of several transfer functions: $LuxA\ = \frac{{Arab/{K_{Arab}}}}{{1 + Arab/{K_{Arab}}}}$, $LuxB\ = \frac{{AHL/{K_{AHL}}}}{{1 + AHL/{K_{AHL}}}}$, and $Out\ = \frac{{LuxA \cdot LuxB}}{{{K_{AB}}}}$, where ${K_{AB}}$ is binding dissociation constant. (**E**) The *luxA-luxB* splitting design. LuxB is regulated by the P_lux_ promoter located on a MCP. LuxR is regulated by a constitutive promoter located on a LCP and is induced by AHL. LuxA is regulated by the P_BAD_ promoter located on a MCP. AraC is regulated by a constitutive promoter located on a LCP and is induced by Arabinose. The *luxCDE genes* are regulated by a constitutive promoter located on an HCP. (**F**) The measured AHL/Arabinose transfer function and bioluminescence output for an AND gate based on the LuxA-LuxB interaction. (**G**) Performance comparison of the two logic AND gates. The output dynamic range (ODR) was calculated as the lowest value of the logarithmic transform of the ratio between the ‘1’ levels and ‘0’ levels: $ODR\ = \ min\left\{ {\log \frac{{{I_{[ {1,1} ]}}}}{{{I_{[ {0,0} ]}}}},\log \frac{{{I_{[ {1,1} ]}}}}{{{I_{[ {0,1} ]}}}},{\rm{\log}}\frac{{{I_{[ {1,1} ]}}}}{{{I_{[ {1,0} ]}}}}}\right\}.$All experimental data represent the average of three experiments. The average and errors (s.e.m.) shown in the figures are derived from three experiments.

In addition to using the *luxCDABE* cassette as a reporter, we modified its gene structure to build circuits that can perform logic computations. To this end, the *luxCDABE* cassette was split into two sub-circuits that were regulated by two different input chemicals. The first sub-circuit received Arabinose and regulated the *luxAB* genes involved in the forward reaction, and the second sub-circuit received AHL and regulated the *luxCDE* genes involved in the reverse reaction (Figure 3A). The transcription factor AraC is produced by a constitutive promoter, located on a LCP. Arabinose binds to AraC, forming a complex which activates the P_BAD_ promoter. LuxR is regulated by a constitutive promoter located on a LCP and is induced by acyl-homoserine-lactone (AHL) to activate P_lux_. The P_BAD_ and P_lux_ promoters, located on a medium-copy-number plasmid (MCP) and an HCP respectively, regulate the luciferase and the complex enzymes required to produce bioluminescence. Re-arranging Equation (7) shows that the bioluminescent signal can compute the minimum between $\frac{{{E_F}}}{{{K_R}}}$ and $\frac{{\ {E_R}}}{{{K_F}}}$ levels $\left(I \propto MIN \left\{{\frac{{E_F}}{{K_R}},\frac{{E_R}}{{K_F}}}\right\}\right)$, (see Section 3 of the Supplementary Data for additional information). Minimum function, which receives continuous physical inputs and displays a vague output over an output dynamic range, is widely used in fuzzy logic to implement conjunction (37). Typically, when the inputs are ‘0’ and ‘1’, the MIN fuzzy lattice acts as an AND Boolean gate (Figure 3B). Given that the bioluminescent enzymes were regulated by inducible promoters which convert the continuous levels of Arabinose and AHL to two-discrete states (‘0’, ‘1’), the design of the MIN fuzzy lattice (Figure 3A) can also implement an AND logic gate (Figure 3C).

The implementation of AND logic gates in living cells often employs the interaction of two cooperative components such as tRNA and mRNA (29), protein-protein interactions (38), or protein-DNA interactions (39). The output of such circuits is activated only when both components are produced. On the other hand, we created an AND logic gate that exploits the paradoxical components which are already present in the biochemical reactions, by controlling independently the forward and reverse rates using two different inputs. To compare the performance of the two designs, we built an AND logic gate based on the LuxA–LuxB protein interaction (Figure 3D). Our strategy was to split the *luxAB* into two parts that are regulated by two different inputs (Figure 3E). The *luxA* gene is regulated by the P_BAD_ promoter located on a MCP which receives Arabinose, and the *luxB* gene is regulated by the P_lux_ promoter located on a MCP which receives AHL. Both AraC and LuxR are produced by a constitutive promoters. The *luxCDE* genes are constitutively produced and located on an HCP. The measured transfer function showed that when both *luxA* and *luxB* were induced by high AHL and Arabinose, a high bioluminescent signal was achieved (Figure 3F). In cases where only one of the *luxA* or *luxB* components was induced at high levels, a low bioluminescence signal was achieved. This transfer function can be approximated as an AND logic gate with an output dynamic range of 0.32 orders of magnitude (Figure 3G). However, it has a poor performance compared to the first design, which is based on the *luxAB-luxCDE* interaction, with an output dynamic range of more than one order of magnitude (Figure 3G). These results can be described by a minimal biochemical model (Figure 3B and D), which incorporates the promoter activities and bioluminescent signals (Equation 7). For simplicity, we assumed that all the promoters are identical and their activity can be described by the Michaelis Menten equation $\left({\frac{x}{{1 + x}}}\right)$. Indeed, AND logic gates that exploit a MIN fuzzy lattice (Figure 3B) showed a better classification with a narrower dynamic range of logic states compared to the AND logic gate that is based on protein-protein interactions (Figure 3D). Further analysis that compared the performance of the two AND logic gates is provided in Section 4 of the Supplementary Data (Supplementary Figures S4–S6).

**Figure 4.**
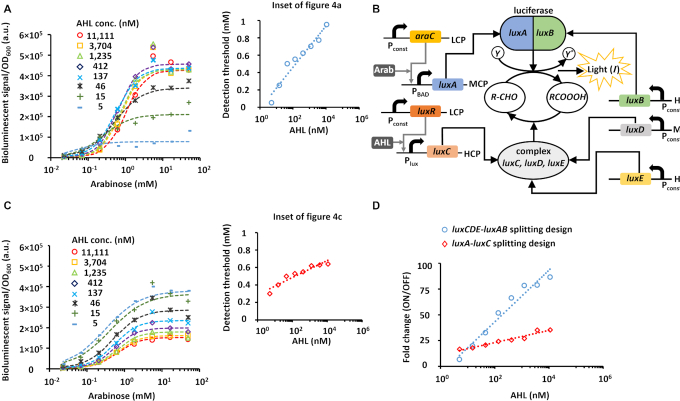
Design of a synthetic comparator with a programmable detection threshold in living cells by splitting the *luxCDABE* cassette. (**A**) Measured transfer function between Arabinose input concentration and bioluminescent signal, for varying acyl-homoserine-lactone (AHL) levels. These data were extracted from Figure 3C. The dots represent the experimental results and the dashed lines fit Hill function curves (see Section 2, Supplementary Table S5 of the Supplementary Data for parameters used for fitting). The inset shows the relationship between the detection threshold (${K_{deff}}$) and AHL concentration. ${K_{deff}}$ was calculated as the input value corresponding to half of the output value. (**B**) Design by further splitting the *luxCDABE* cassette. The *luxC* is regulated by the P_lux_ promoter located on a high copy number plasmid (HCP). LuxR is regulated by a constitutive promoter located on a low copy number plasmid (LCP) and is induced by AHL to activate P_lux_. The *luxA* is regulated by the P_BAD_ promoter located on a medium copy number plasmid (MCP). AraC is regulated by a constitutive promoter located on a LCP and is induced by Arabinose to activate P_BAD_. The *luxD* is regulated by a constitutive promoter located on MCP. The *luxB* and *luxE* are regulated by a constitutive promoter located on an HCP. (**C**) Measured transfer function between Arabinose input concentration and bioluminescence signal for varying AHL levels. The dots represent the experimental results and the dashed lines fit Hill function curves (see Section 2, Supplementary Table S5 of the Supplementary Data for parameters used for fitting). The inset shows the relationship between the detection threshold (${K_{deff}}$) and AHL concentrations. The ${K_{deff}}$ was calculated as the input value corresponding to half of the output value. (**D**) Fold change comparison (ON/OFF ratio) for *luxCDE-luxAB* (data based on A, Supplementary Table S6) and the new design by further *luxA-luxC* splitting of the *luxCDABE* cassette (data based on C, Supplementary Table S7). All experimental data represent the average of three experiments. Further statistical analysis is provided in Section 2 of the Supplementary Data.

Genetic circuits with tunable detection thresholds (Figure 1B) can be used to discretize continuous input values into multiple distinct outputs, acting as comparators (30,35). Such circuits have recently attracted widespread attention (25) because they can detect graded environmental inputs, and reliably report outputs with two states. The measured signals of biosensors are often encoded by two logic states instead of analog levels, in order to tolerate noise and compensate for distortion of biological signals. Consequently, combining comparators with detection thresholds ranging from low to high levels, results in a system that can convert graded environmental signals to digital signals with high sensitivity. In such a case, only comparators that have detection thresholds lower than the input level are activated and display high outputs (Section 5, Supplementary Figure S7 of the Supplementary Data). Circuits with programmable detection thresholds are also useful for biological systems where the detection threshold is mismatched with application and design requirements (19,40). As described above, controlling the reverse reaction in the *luxCDABE* cassette independently from the forward reaction (Figure 3A), allowed us to build a system with a programmable detection threshold using AHL (Figure 4A, Supplementary Figure S2). The estimated detection threshold of the transfer function increases as AHL concentrations increase (inset of Figure 4A). However, the experimental results showed that this design also affected the bioluminescence signal, when AHL concentrations changed. To reduce this dependency, we optimized the expression level of the lux enzymes by a further splitting of the operon. In the new circuit, only *luxA* and *luxC* were controlled by inducible promoters (P_BAD_/Arabinose, P_lux_/AHL, respectively), while *luxB, luxD*, and *luxE* were controlled by constitutive promoters (Figure 4B). The new strategy resulted in a transfer function with a programmable detection threshold (Figure 4C, Supplementary Figure S3), that increased with AHL concentration (Inset of Figure 4C), while the fold change of the ON/OFF ratio was well maintained in contrast to the first design (Figure 4D, Supplementary Tables S6, S7).

**Figure 5.**
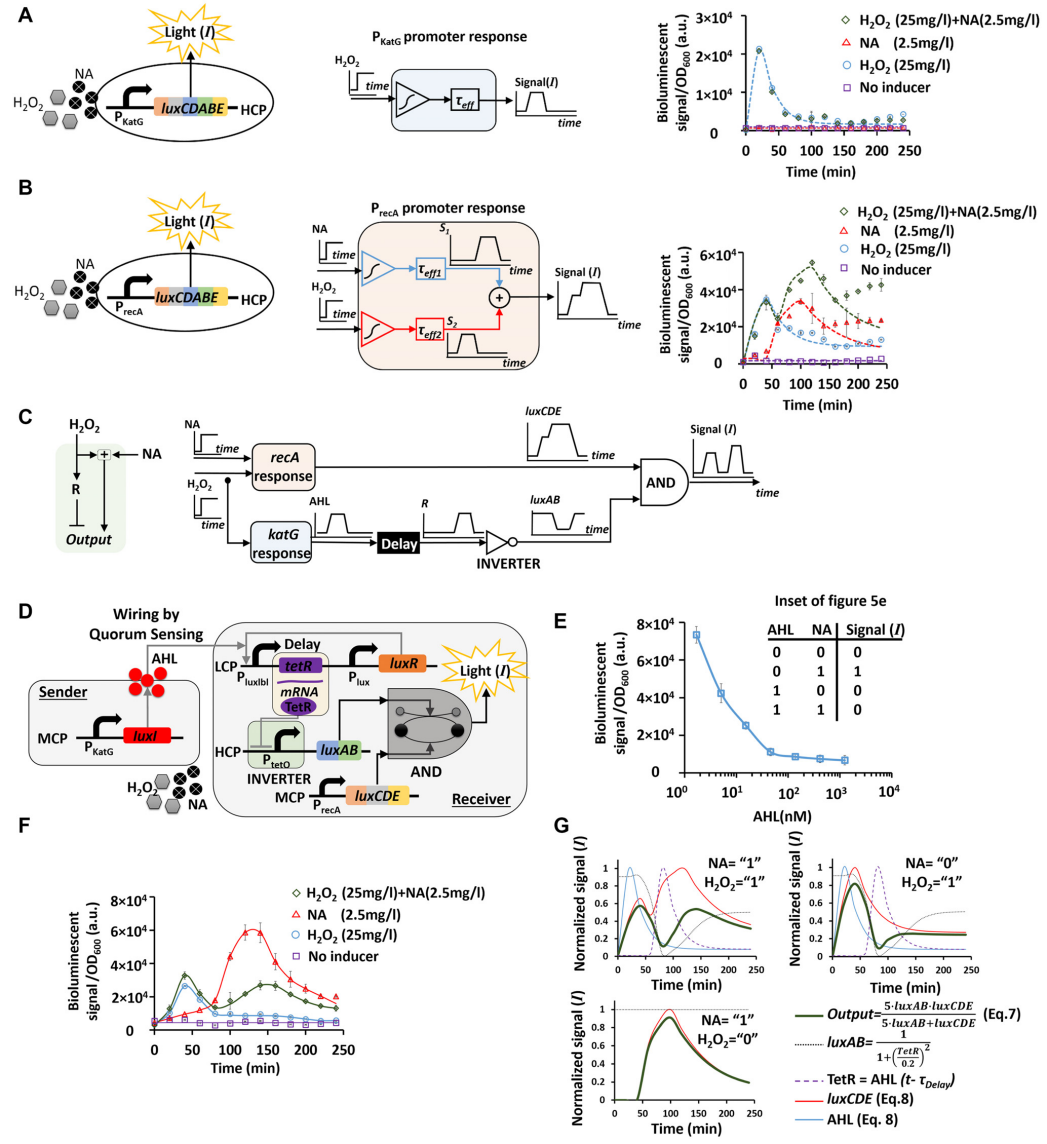
A synthetic crosstalk-compensating circuit for bacterial biosensors. (**A**) A synthetic gene circuit (left) and schematic model (center) for *katG*-based bacterial biosensor. Time courses (right) of *katG* promoter in response to 25 mg/l of peroxide (H_2_O_2_) and 2.5 mg/l of nalidixic acid (NA), alone and in combination. The dots represent experimental results and the dashed lines are simulation results (Equation 8) with simulation parameters: *τ_1_*= 20 min, *τ_eff_*= 20 min, *τ_D_*_1_ = 0 min, *τ_D2_*= 0 min, *β* = 0.02, *c* = 2000 (see Section 2, Supplementary Table S9 of the Supplementary Data for parameters used for fitting). (**B**) A synthetic gene circuit (left) and schematic model (center) for *recA*-based bacterial biosensor. Time courses (right) of *recA* promoter in response to 25 mg/l of H_2_O_2_ and 2.5 mg/l of NA, alone and in combination. The dots represent experimental results and the dashed lines are simulation results (Equation 8). Simulation parameters for the fitting of NA response were *τ_1_*= 60 min, *τ_eff_*= 25 min, *τ_D1_*= 60 min, *τ_D2_*= 100 min, *β* = 0.1, *c* = 9000. Simulation parameters for the fitting of H_2_O_2_ response were *τ_1_*= 30 min, *τ_eff_*= 30 min, *τ_D1_*= 0 min, *τ_D2_*= 40 min, *β* = 0.25, *c* = 9000 (see Section 2, Supplementary Table S9 of the Supplementary Data for parameters used for fitting). (**C**) A schematic model of the crosstalk-compensating circuit. The model consists of *recA* and *katG* promoters, and an incoherent type-1 feedforward loop, which is built from a time-delay, a repressor, and an AND logic gate. (**D**) The crosstalk-compensating circuit is segmented into ‘sender’ and ‘receiver’ parts carried by two bacterial strains. Acyl-homoserine-lactone (AHL) quorum-sensing molecules were used to wire between the sender and the receiver. The sender included the *katG* promoter that regulates LuxI located on a MCP to produce AHL. There are three plasmids in the receiver. On the LCP, the promoter P_luxlbl_ (which is activated by the AHL-LuxR complex and has a very low basal level (10)) regulates the expression of TetR, and the promoter P_lux_ regulates the expression of LuxR. On the MCP, the promoter *recA* regulates the expression of the *luxC, luxD* and *luxE*. On the HCP, the P_tetO_ promoter regulates the expression *of* the *LuxA and LuxB*. AHL produced from the sender binds LuxR and activates P_lux_ and P_luxlbl_. The TetR repressor is located on the LCP and binds P_tetO_ located on the HCP, inhibiting the activity of luciferase. (**E**) Measured transfer function of the receiver circuit using externally added AHL. The inset shows NIMPLY logic gate (NOT-IMPLY) that is implemented by the receiver circuit with externally supplied AHL. (**F**) Time courses of the crosstalk-compensating circuit response in the presence of 25 mg/l of peroxide (H_2_O_2_) and 2.5 mg/l of nalidixic acid (NA) alone and in combination. The sender and receiver circuits carried by two different bacterial strains were mixed together at a ratio of 1 to 10, respectively. (G) Simulated time courses of proteins production by the crosstalk-compensating circuit in the presence of NA or H_2_O_2_ or both. The average and errors (s.e.m.) shown in the figures are derived from three experiments.

To demonstrate the applicability of the proposed work, we used the MIN fuzzy lattice to reduce the unwanted crosstalk amongst bacterial biosensors and improve the detection specificity (i.e. a biosensor that is active for a specific chemical substance). The low detection specificity of environmental stress-responsive promoters (41) is considered to be a major challenge in the development of whole-cell bacterial biosensors. Here, we built two bacterial biosensors that can detect peroxide (H_2_O_2_) and nalidixic acid (NA) by fusing the *luxCDABE* reporter with *katG* and *recA* stress-responsive promoters, respectively (Figure 5A, B). The *katG* promoter is activated by oxidative stress in response to intracellular production of oxygen radicals (28), and the *recA* promoter is activated upon DNA damage (27). A simple kinetic model of stress-responsive promoters assumed that: (i) proteins that are regulated by stress-responsive promoters often have effective half-lives (${\tau _{eff}}$), and their expression level is proportional to the initial concentration of chemical substances (${X_0}$); (ii) chemical substances (H_2_O_2_ and NA) can be rapidly consumed by the bacteria in time ${\tau _1}$ and (iii) chemical chain reactions which are involved in the *recA* stress promotion causes a response time delay in the consumption processes of NA and H_2_O_2_ (${\tau _{D1}}$ and ${\tau _{D2}}$*_,_*respectively) (see Section 2, Supplementary Table S8 of the Supplementary Data for list of parameters). Furthermore, the process of generating light by the *luxCDABE* cassette is faster than the activation of the *recA* and *katG* promoters. Therefore, we assumed that the process of light generation is at steady state and is given by:

(8) $$\begin{equation*}I(t) \propto {X_0} \cdot \left( {1 - {e^{ - \frac{{t - {\tau _{D1}}}}{{{\tau _{eff}}}}}}} \right) \cdot \left( {{e^{ - \frac{{t - {\tau _{D2}}}}{{{\tau _1}}}}} + \beta } \right) + c,\end{equation*}$$

where $\beta$ is the basal level concentration of the chemical substances (e.g. NA and H_2_O_2_) that cannot be degraded by the bacteria and is always present in the culture, and *c* is the basal level of the bioluminescence. In Equation (8) we assumed that ${e^{ - \frac{{t - {\tau _{Di}}}}{{{\tau _1}}}}} = \ 1,{\rm{\ when}}\ t < {\tau _{Di}}\ for\ i\ = \ 1,2$. The bacterial biosensors were exposed to H_2_O_2_, NA or a mixture of both. Our experimental results showed that the bioluminescent signal of *katG*-based bacterial biosensor was highly specific to H_2_O_2_ (Figure 5A). However, the bioluminescent signal of the *recA*-based bacterial biosensor responded to both H_2_O_2_ and NA (Figure 5B). The experimental results of *katG* and *recA* matched our empirical model (Equation 8), where the non-specificity of *recA* for H_2_O_2_ and NA was modelled by a summation between NA and H_2_O_2_ responses (Figure 5B). While the time response of *recA* showed two peaks that are related to the two different chemicals, it is challenging to use this information in real world applications. This is because that the first peak, related to H_2_O_2_, has a small magnitude, and could disappear as a result of environmental changes and random fluctuations (see Section 6, Supplementary Figures S8 and S9, Supplementary Table S10 of the Supplementary Data). To improve the performance of the *recA* promoter, we designed a circuit, which we termed crosstalk-compensating, that can reliably correlate the number of bioluminescent peaks to the number of chemical types (Figure 5C). For example, the crosstalk-compensating circuit based on r*ecA* would display two clearly separated peaks when both NA and H_2_O_2_ are present in the culture. The circuit used the *recA* promoter to activate the output by both NA and H_2_O_2_, and at the same time, used *katG* to repress the output by H_2_O_2_ only. The duality of H_2_O_2_ can be achieved by using a repressor ($R$) that is regulated by the *katG* promoter (Figure 5C). Accordingly, while the first input of the AND gate (*luxCDE*) is connected directly to *recA*, the second input (*luxAB*) is connected to *katG* via a repressor which leads to a delay in its response. If the time delay is synchronized with the kinetics of NA response, two separated peaks can be achieved. The design principle underlying the proposed circuit is analogous to an incoherent type-1 feedforward loop (42) using two parallel antagonistic paths of the AND operation.

The crosstalk-compensating circuit was further segmented into ‘sender’ and ‘receiver’ circuits carried by two different bacterial strains, using quorum sensing molecules (AHL) for wiring (43) (Figure 5D). The sender circuit specifically detected H_2_O_2_ using the *katG* promoter and produced AHL molecules by regulating LuxI protein expression. The receiver circuit collected the diffusing AHL molecules and expressed the TetR repressor, which was regulated by P_lux_ and LuxR. The receiver circuit was comprised of an AND gate, which was implemented by the *luxAB-luxCDE* interaction, where the *luxCDE* was activated by the *recA* promoter and *luxAB* were regulated by P_tetO_ and repressed by TetR. The delay between the *recA* and *katG* responses was programmed by the kinetics of TetR expression and the dynamics of AHL diffusion. Initially, we tested the receiver circuit with externally supplied AHL (Figure 5E). The measurements taken at the steady state showed that a high bioluminescent signal was measured when the AHL concentration was low, and vice versa. This circuit can also act as NOT IMPLY logic gate (inset Figure 5E). Then, we tested the sender and receiver circuits carried by two different strains, at various ratios of the strains. The optimal ratio was one volume of cells carrying the sender circuit to ten volumes of cells carrying the receiver circuit. The experimental results indicated that the number of measured peaks for the bioluminescent signal was proportional to the number of types of chemicals (Figure 5F). For example, two separated peaks were measured when NA and H_2_O_2_ were present in the culture. We also built a simplified biochemical model that thoroughly captured the kinetics of the crosstalk-compensating circuit (Figure 5G). The model was based on Equations (7 and 8), using a consistent set of model parameters. The time delay (${\tau _{Delay}}$) between the two parallel antagonistic regulation paths of the *luxCDABE* cassette was incorporated into the kinetics of TetR (TetR$ \propto$AHL$( {t - {\tau _{Delay}}} )$) and the *luxAB* level was proportional to P_tetO_ activity $\left({luxAB} = \frac{1}{{1 + {{\left({\frac{{TetR}}{{0.2}}}\right)}^2}}}\right)$. Further analysis that compared the performance of the *recA*-based bacterial biosensor and the crosstalk-compensating circuit, including stochastic behaviour, is provided in Section 7 (Supplementary Figure S10) of the Supplementary Data. In conclusion, while bacterial biosensors have a slow response compared to chemical sensors, they can be programmed to provide sensitivity for a very wide-range of chemicals and analytes.

## DISCUSSION

Integration of real-world physical conditions (*i.e*., stress response, chemotaxis, metabolic stimulus, cell-to-cell communication, temperature and pH level) with minimal systems in the framework of synthetic biology, will be necessary to bring bacterial biosensors to the next level. In synthetic minimal systems, we optimize the trade-offs among reliability, resource usage, number of synthetic parts, and protein expression levels. In an effort to reveal design principles underlying such systems, we created biological circuits that integrate transcriptional regulation of gene expression with metabolic pathways. Specifically, we redesigned the *luxCDABE* cassette by splitting the five genes into different combinations to execute sophisticated analog and digital computational functions (e.g. MIN fuzzy lattice soft minimum, which finds the minimum value between two analog signals). Minimum functions are widely used in fuzzy logic computation to implement conjunction (44) and when they are combined with inhibition, they can act as a universal gate for processing real-world bio-signals.

Compared to the implementation of the recently proposed AND logic gates in living cells (29), which rely on the cooperative binding of protein-protein interaction, our AND logic gate asymptotically approximates the soft minimum as a logical conjunction. This was achieved by exploiting paradoxical elements which are already naturally present in biochemical reactions, such as controlling the forward and reverse rates using independent inputs. The proposed AND logic gate exhibited a higher fold change and narrower input dynamic range between logic states compared to AND logic gates that are based on protein-protein interactions. We also built a comparator with a programmable detection threshold, which can convert continuous information into discrete levels. Genetic comparators may be beneficial for bio-sensing applications, by allowing robust measurement of stress-environmental signals. One advantage of our circuit in comparison to state-of-the-art biological circuits with programmable detection thresholds (e.g. two-component system (25) and recombinase-based circuits (30)), is the ability to program both the threshold and the saturation level allowing us to carry out further complex computations. Furthermore, our circuits can incorporate complex temporal dynamics such as the cross-talk compensating circuit (Figure 5D).

Finally, we used the genetically engineered *luxCDABE* cassette to build a crosstalk-compensating circuit that improves the performance of bacterial biosensors. The proposed circuit reduced the unwanted crosstalk between chemical substances and stress-responsive promoters (*recA* and *katG*) and generated information that is directly proportional to the number of different substances in the culture. The new circuit was based on implementing several engineering concepts into bacterial biosensors, such as an incoherent feedforward loop and mixed signals processing that combine analog processing and decision-making. We demonstrated that the engineered *luxCDABE* cassette can be applied to several inducible promoters (e.g. P_BAD_ and P_lux_) and stress-responsive promoters (e.g. *recA* and *katG*). We also expect that our splitting strategy can be implemented in optimizing *luxCDABE* cassette for either high-GC bacteria (45) or mammalian cells (46). This flexibility can address technological challenges and broaden the range of industrial, diagnostic and biomedical applications.

## Supplementary Material

gkz807_Supplemental_File
