# Supplementary material for: Synthetic metabolic computation in a bioluminescence-sensing system
Source: Nucleic Acids Res. 2019 Sep 23;47(19):10464–74. doi: 10.1093/nar/gkz807 (PMC6821183; doi:10.1093/nar/gkz807)
Supplement: gkz807_Supplemental_File [file gkz807_supplemental_file.pdf]

Supplementary Data for

Synthetic metabolic computation in a bioluminescence-sensing system

Natalia Barger[1], Phyana Litovko[1], Ximing Li[1], Mouna Habib[1], and Ramez Daniel[1*].

Correspondence to: ramizda@bm.technion.ac.il

This PDF file includes:

Supplementary Text

Supplementary Figures S1 to S10

Supplementary Tables S1 to S11

Table of Contents

Section 1- Materials and Methods 2

Supplementary Table S1 The genotype of bacterial strains 2

Supplementary Table S2 List of primers includes the restrictions sites sequence 2

Supplementary Figure S1 Plasmid maps 3

Supplementary Table S3 Plasmid combinations 4

Section 2- Modeling and Fitting 5

2.1 Designing and Modeling of a Bioluminescent *luxCDABE* Cassette 5

Supplementary Table S4 List of parameters for modeling 5

Supplementary Table S5 The parameters used for fitting of transfer functions 6

2.2 Further statistical analysis of measured transfer function between Arabinose input concentration and bioluminescent signal 6

Supplementary Figure S2 Measured transfer function between Arabinose input concentration and bioluminescent signal for *luxCDE-luxAB* splitting design. 7

Supplementary Table S6 Measured bioluminescent signals and Fold change values for *luxCDE-luxAB* splitting design 7

Supplementary Figure S3 Measured transfer function between Arabinose input concentration and bioluminescent signal for further *luxA-luxC* splitting design. 8

Supplementary Table S7 Measured bioluminescent signals and Fold change values for further *luxA-luxC* splitting design 8

2.3 The kinetic model of stress-responsive promoters 9

Supplementary Table S8 List of parameters for kinetic model 9

Supplementary Table S9 The parameters used for stress promoters time courses fitting 9

Section 3 - MIN fuzzy lattice by the *luxCDABE* cassette 9

Section 4 - Analysis of AND logic gates performance 10

Supplementary Figure S4 Simulation results of fold change as a function of input dynamic range. 12

Supplementary Figure S5 Simulation results of AND logic gates 13

Supplementary Figure S6 Simulation results of AND logic gates represented as logic states 13

Section 5 - Programmable detection threshold circuit 14

Supplementary Figure S7 Biosensors that behave in a digital manner with programmable detection thresholds 15

Section 6 - Further comparison of the *recA*-based bacterial biosensor and crosstalk-compensating circuit performance 16

Supplementary Figure S8 Bioluminescent signal at time points following *recA* promoter induction includes H_2O_2 peak magnitude (H_1) and the maximal standard deviation (σ_{max}). 16

Supplementary Figure S9 The performance of the *recA*-based bacterial biosensor and crosstalk-compensating circuit for various H_2O_2 and NA concentrations 17

Supplementary Table S10 The magnitude of peaks for various NA and H_2O_2 concentrations 17

Section 7 Stochastic behavior of bacterial biosensors 18

Supplementary Figure S10 Stochastic simulation of bacterial biosensors. 19

Section 8 Biological parts 20

Supplementary Table S11 List of biological parts 20

Supplementary References 24

Section 1- Materials and Methods

Supplementary Table S1 The genotype of bacterial strains

Escherichia coli (*E. coli*) 10 β	araD139 D (ara-leu) 7697 fhuA lacX74 galK (W80 D (lacZ) M15) mcrA galU recA1 endA1 nupG rpsL (StrR) D (mrr-hsdRMS-mcrBC)
E. coli MG1655	F^{-} λ^{-} $ilvG^{-}$ rfb-50 rph-1

Supplementary Table S2 List of primers includes the restrictions sites sequence

Gene	Primer
***kpnI**-LuxA-f*	ca **GGT ACC** ATG AAA TTT GGA AAC TTT TTG CTT AC
***xmaI**-LuxA-r*	taat **CCCGGG** CTAATATAATAGCGAACGTTGTT TTT
***kpnI-**LuxB-f*	cac **GGTACC** ATGAAATTTGGATTGTTCTTCCTTAA
***xmaI**-LuxB-r*	taat **CCCGGG**TTAGGTATATTCCATGTGGTACTT
***kpnI**-LuxC-f*	cac **GGTACC** ATGACTAAAAAAATTTCATTCATTATTAACG
***xmaI**-LuxC-r*	cataat **CCCGGG** TTATGGGACAAATACAAGGAACTT
***kpnI**-LuxD-f*	caca **GGTACC** ATGGAAAATGAATCAAAATATAAAACCAT
***xmaI**-LuxD-r*	taatca **CCC GGG** TTA AGA CAG AGA AAT TGC TTG ATT TT
***kpnI**-LuxE-f*	cacac **GGT ACC** A TGA CTT CAT ATG TTG ATA AAC AAG AA
***xmaI**-LuxE-r*	taatca **CCC GGG** TCAACTATCAAACGCTTCGGTT
***AatII**-katG-f*	ta **GACGTC** CGAAATGAGGGCGGGAAA
***kpnI** -katG-r*	ca **GGTACC** TTTCTCCTCTTTAATAACGTTGCTGACCACGACCC
***AatII**-recA-f*	ttaa **GACGTC** AGAGAAGCCTGTCGGCAC
***kpnI**-recA-r*	ttc **GGTACC** CGCTTTCTGTTTGTTTT

Supplementary Figure S1 Plasmid maps

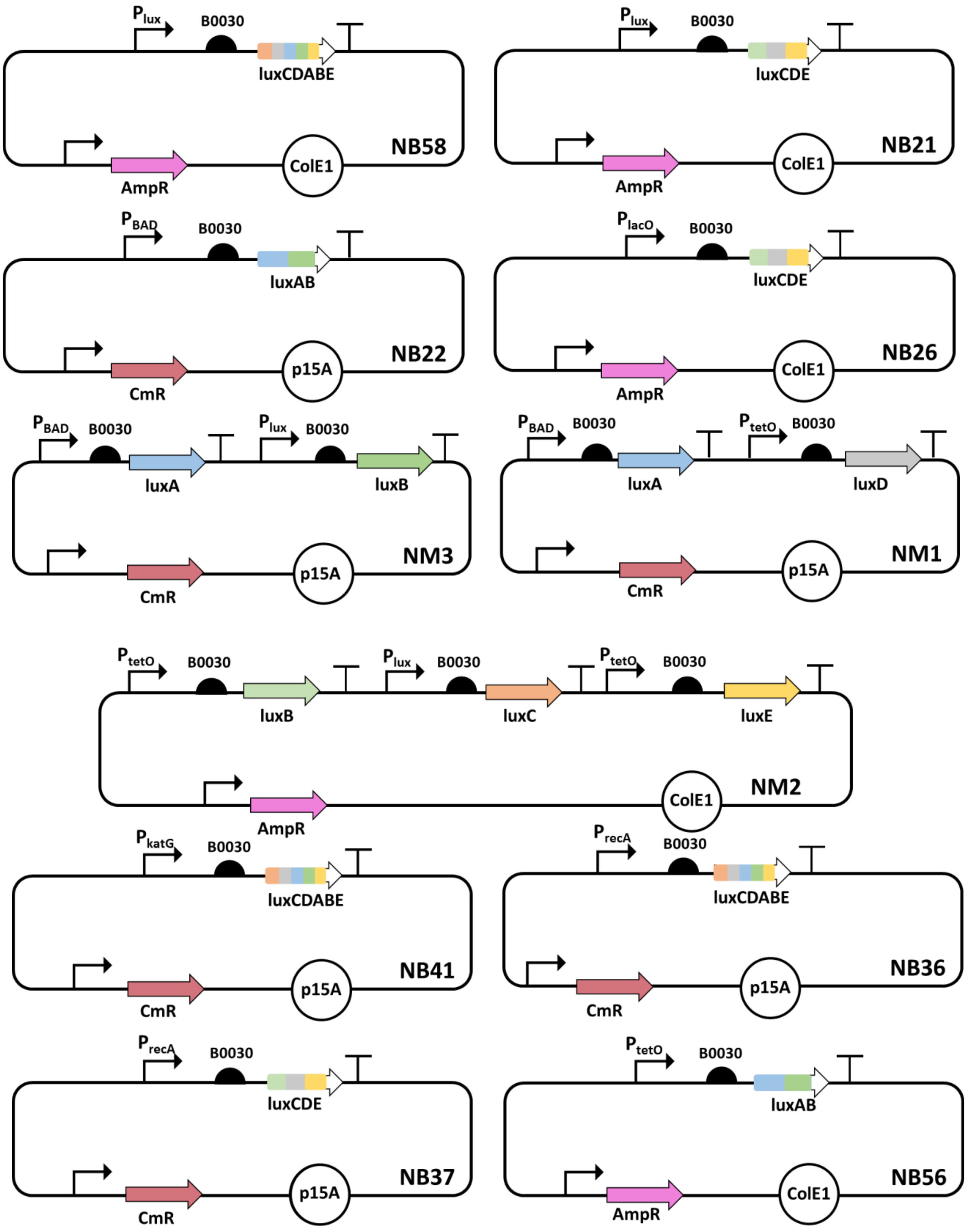

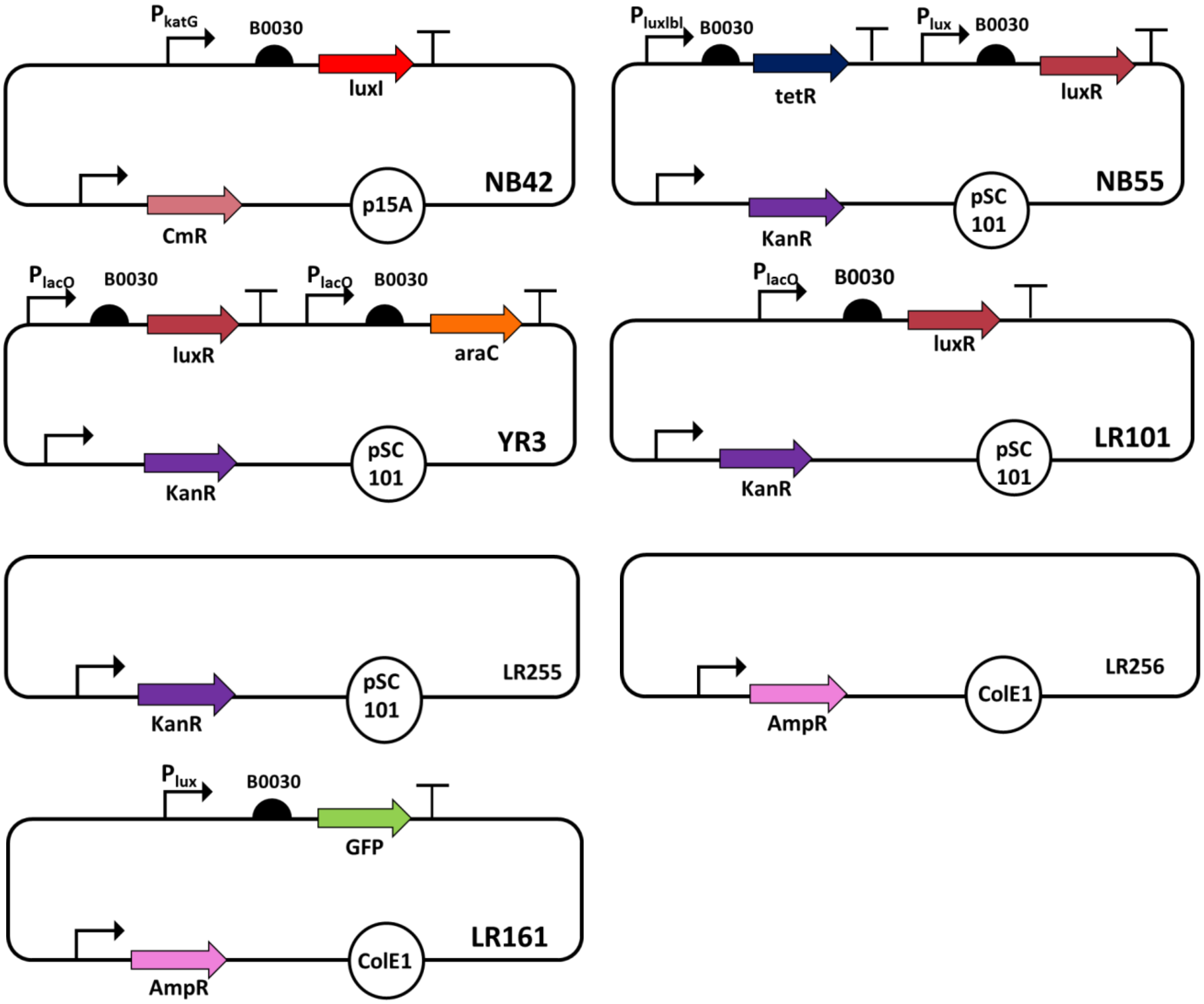

Supplementary Table S3 Plasmid combinations

Plasmids	Figures
LR101+NB58	Fig. 2a, Fig. 2b
LR101+LR161	Fig.2b
YR3+NB21+NB22	Fig.3a,Fig.3c,Fig.4a, Fig.4d
YR3+NB26+NM3	Fig.3e, Fig.3c
YR3+NM1+NM2	Fig. 4b, fig. 4c, Fig. 4d
NB41+LR255+LR256	Fig. 5a
NB36+LR255+LR256	Fig. 5b
NB42+LR255+LR256,NB37+NB55+NB56	Fig. 5d, Fig 5f
NB37+NB55+NB56	Fig. 5e

Section 2- Modeling and Fitting

2.1 Designing and Modeling of a Bioluminescent *luxCDABE* Cassette

Supplementary Table S4 List of parameters for modeling

Symbol	Description
$R-COOH$	Fatty acid
$R-CHO$	Long-chain aliphatic aldehyde
$FMNH_2$	Reduced flavin mononucleotide
FMN	Flavin mononucleotide
O_2	Oxygen
H_2O	Water
ATP	Adenosine triphosphate
AMP	Adenosine monophosphate
$NADPH$	Nicotinamide adenine dinucleotide phosphate molecules in reduced form
$NADP^+$	Nicotinamide adenine dinucleotide phosphate molecules in oxidized form
PP	Diphosphate
E_F	Luciferase expression level
E_R	Complex enzymes expression level
K_F	Rate of the forward reaction which is catalyzed by luciferase
K_R	Rate of the reverse reaction which is catalyzed by reductase and synthetase
S	Concentration of long-chain aliphatic aldehyde
P	Concentration of long-chain aliphatic aldehyde
Y	Concentration of reduced flavin mononucleotide
Y^*	Concentration of flavin mononucleotide
S_T	Total concentration of long-chain aliphatic aldehyde
Y_T	Initial concentration of reduced flavin mononucleotide
K_{deff}	Detection threshold that depends on the level of complex enzymes
n	Hill coefficient
β	Basal level
I	Bioluminescent signal

Supplementary Table S5 The parameters used for fitting of transfer functions

AHL concentrations (nM)	*The luxCDE-luxAB* spitting (Fig. 4a)			Further *luxA-luxC* splitting (Fig. 4c)		
	K_{deff}	n	β	K_{deff}	n	β
11,111	0.95	1.67	0.01	0.64	1.5	0.01
3,704	0.77	1.67	0.01	0.63	1.5	0.01
1,235	0.70	1.67	0.01	0.62	1.5	0.01
412	0.62	1.67	0.01	0.55	1.5	0.01
137	0.55	1.5	0.01	0.53	1.4	0.01
46	0.50	1.2	0.01	0.50	1.2	0.01
15	0.25	0.9	0.01	0.40	0.9	0.01
5	0.05	0.9	0.01	0.30	0.9	0.01

2.2 Further statistical analysis of measured transfer function between Arabinose input concentration and bioluminescent signal

This section provides further statistical analysis of the experimental data in Fig. 4a and Fig. 4c in the main text. Figure S2 represents the average values and errors (s.e.m.) from three experiments for *luxCDE-luxAB* splitting design (Fig.3a) for varying acyl-homoserine-lactone (AHL) levels. Figure S3 represents the average values and errors (s.e.m.) from three experiments for further *luxA-luxC* splitting design (Fig. 4b) for varying AHL levels.

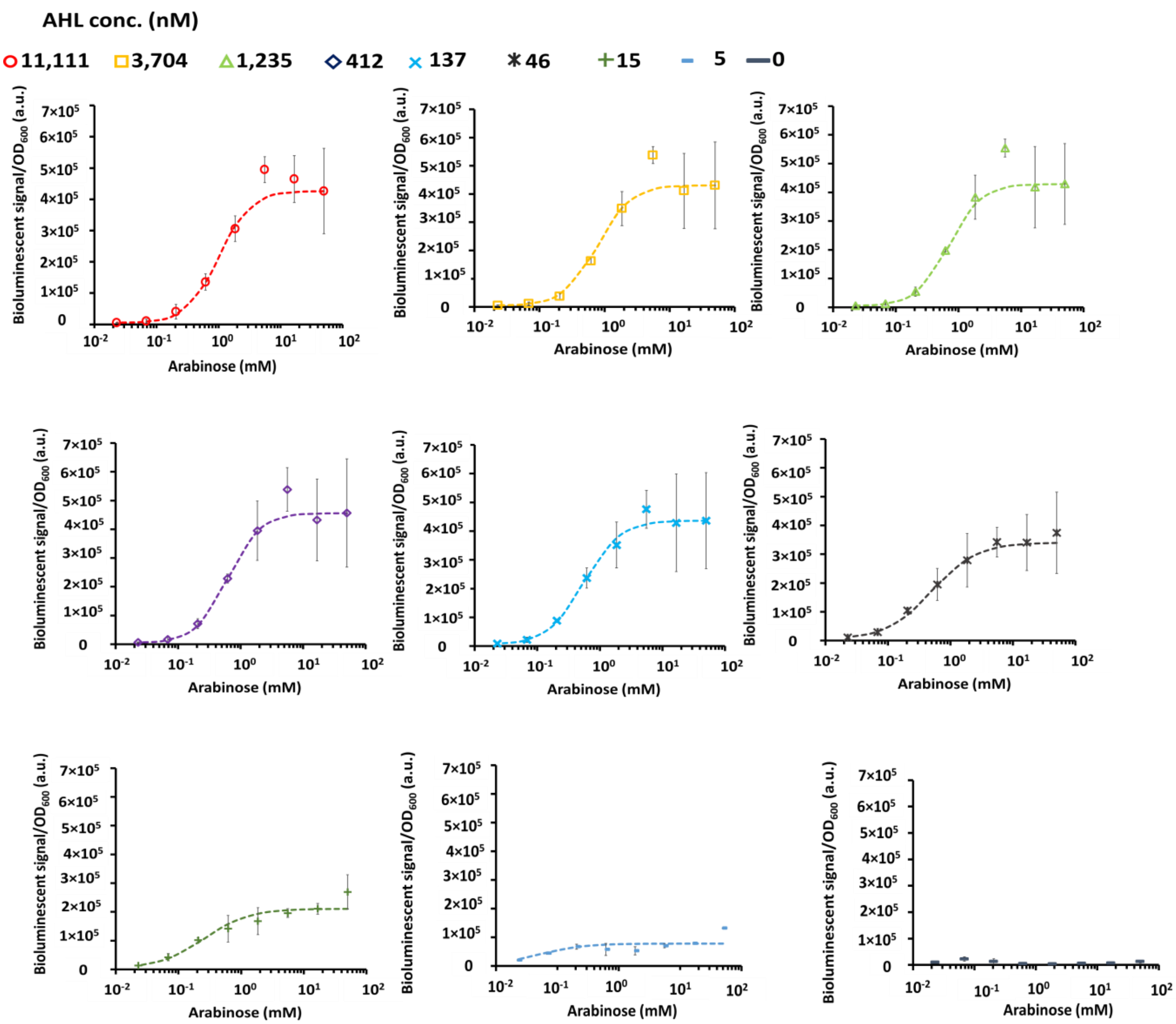

Supplementary Figure S2 Measured transfer function between Arabinose input concentration and bioluminescent signal for *luxCDE-luxAB* splitting design. The dots represent the experimental results and the dashed lines fit Hill function curves.

Supplementary Table S6 Measured bioluminescent signals and Fold change values for *luxCDE-luxAB* splitting design

	**AHL, nM**							
**Arabinose ,mM**	**11,111**	**3,704**	**1,235**	**412**	**137**	**46**	**15**	**5**
High	426,324	430,783	429,122	456,570	436,400	374,525	268,996	131,103
Low	4,926	5,464	5,473	6,886	8,445	11,526	13,608	19,903
**Fold change (ON/OFF ratio)**	**87**	**79**	**78**	**66**	**52**	**32**	**20**	**7**

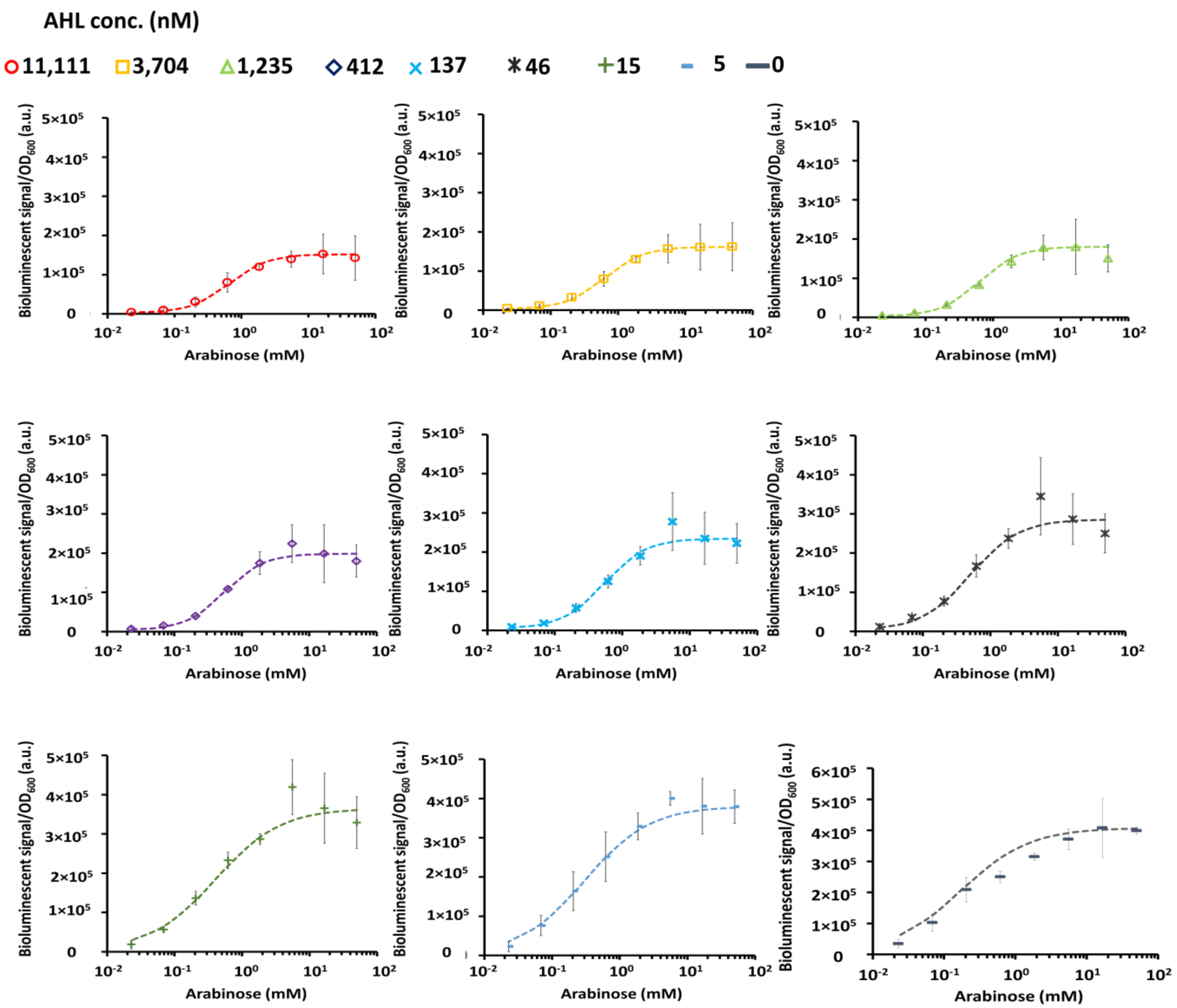

Supplementary Figure S3 Measured transfer function between Arabinose input concentration and bioluminescent signal for further *luxA-luxC* splitting design. The dots represent the experimental results and the dashed lines fit Hill function curves.

Supplementary Table S7 Measured bioluminescent signals and Fold change values for further *luxA-luxC* splitting design

	**AHL, nM**							
**Arabinose ,mM**	**11,111**	**3,704**	**1,235**	**412**	**137**	**46**	**15**	**5**
High	142,760	162,100	150,713	179,930	222,554	250,207	329,192	379,241
Low	4,048	4,615	5,596	7,018	9,182	12,113	18,436	22,897
**Fold change (ON/OFF ratio)**	**35**	**35**	**27**	**26**	**24**	**21**	**18**	**17**

2.3 The kinetic model of stress-responsive promoters

Supplementary Table S8 List of parameters for kinetic model

Symbol	Description
τ_{eff}	Effective half-lives
X_0	Initial concentration of chemical substances
τ_1	Consumption time of chemical substances by the bacteria
$\tau_{D1,2}$	Time delay in promoter response
β	Basal level of the chemical substances that cannot be degraded by the bacteria
c	Basal level of the bioluminescence
τ_{Delay}	The time delay between the two parallel antagonistic regulation paths of the *luxCDABE* cassette

Supplementary Table S9 The parameters used for stress promoters time courses fitting

Symbol	P_{KatG} promoter response (Fig.5a in the main text)	P_{recA} promoter response (Fig.5b in the main text)	
	H_2O_2	H_2O_2	NA
τ_{eff}	20	25	30
τ_1	20	60	30
τ_{D1}	0	60	0
τ_{D2}	0	100	40
β	0.02	0.1	0.25
c	2000	9000	9000

Section 3 - MIN fuzzy lattice by the *luxCDABE* cassette

In the main text we proved that the bioluminescent signal is given by (also Eq. 7 in the main text):

$$I \propto \frac{S_T \cdot Y_T \cdot K_R}{K_d} \cdot E_R \cdot \frac{\frac{E_F}{K_{deff}}}{1+\frac{E_F}{K_{deff}}} \text{, where } K_{deff} \equiv \frac{K_R \cdot E_R}{K_F}. \quad (1)$$

By substituting the last term of K_{deff} in the bioluminescent signal, we get:

$$I \propto \frac{S_T \cdot Y_T \cdot K_R}{K_d} \cdot E_R \cdot \frac{\frac{E_F}{E_R \cdot K_R / K_F}}{1+\frac{E_F}{E_R \cdot K_R / K_F}} \quad (1.1)$$

$$I \propto \frac{S_T \cdot Y_T}{K_d} \cdot \frac{E_F \cdot K_F}{1+\frac{E_F \cdot K_F}{E_R \cdot K_R}} \quad (1.2)$$

$$I \propto \frac{S_T \cdot Y_T}{K_d} \cdot \frac{E_F \cdot K_F}{\left(1+\frac{E_F \cdot K_F}{E_R \cdot K_R}\right) \cdot \frac{E_R}{E_R}} \quad (1.3)$$

$$I \propto \frac{S_T \cdot Y_T}{K_d} \cdot \frac{E_F \cdot K_F \cdot E_R}{\left(E_R+\frac{E_F \cdot K_F}{K_R}\right)} \quad (1.4)$$

$$I \propto \frac{S_T \cdot Y_T}{K_d} \cdot \frac{E_F \cdot K_F \cdot E_R}{\left(E_R+\frac{E_F \cdot K_F}{K_R}\right) \cdot \frac{K_F}{K_F}} \quad (1.5)$$

$$I \propto \frac{S_T \cdot Y_T}{K_d} \cdot \frac{E_F \cdot E_R}{\left(\frac{E_R}{K_F}+\frac{E_F}{K_R}\right)} \quad (1.6)$$

$$I \propto \frac{S_T \cdot Y_T}{K_d \cdot K_F \cdot K_R} \cdot \frac{\frac{E_F}{K_R} \cdot \frac{E_R}{K_F}}{\left(\frac{E_R}{K_F}+\frac{E_F}{K_R}\right)} \quad (1.7)$$

$$I \propto MIN\left\{\frac{E_F}{K_R}, \frac{E_R}{K_F}\right\} \quad (1.8)$$

Section 4 - Analysis of AND logic gates performance

In this section, we compare the performance of the AND logic gate based on the MIN fuzzy lattice and the AND logic gate based on protein-protein (LuxA-LuxB) interaction. The equation that describes the AND logic gate based on the MIN fuzzy lattice is:

$$f(u_1, u_2) = \frac{\frac{u_1}{K_1} \cdot \frac{u_2}{K_2}}{\frac{u_1}{K_1}+\frac{u_2}{K_2}} \quad (1)$$

The function *f* has two asymptotical values: f_L ➔ Low, and f_H ➔ High, which can be achieved when:

$$\frac{u_1}{K_1} \equiv \varepsilon \ll 1, \quad \frac{u_2}{K_2} = \varepsilon \ll 1 \quad \rightarrow f_L = \frac{\varepsilon \cdot \varepsilon}{\varepsilon+\varepsilon} = \frac{\varepsilon}{2} \quad (1.1)$$

$$\frac{u_1}{K_1} = a \cdot \varepsilon \gg 1, \quad \frac{u_2}{K_2} = \varepsilon \ll 1 \quad \rightarrow f_L = \frac{a \cdot \varepsilon \cdot \varepsilon}{a\varepsilon+\varepsilon} = \frac{a}{a+1} \cdot \varepsilon \quad (1.2)$$

$$\frac{u_1}{K_1} = \varepsilon \ll 1, \quad \frac{u_2}{K_2} = a \cdot \varepsilon \gg 1 \quad \rightarrow f_L = \frac{a \cdot \varepsilon \cdot \varepsilon}{a\varepsilon+\varepsilon} = \frac{a}{a+1} \cdot \varepsilon \quad (1.3)$$

$$\frac{u_1}{K_1} = a \cdot \varepsilon \gg 1, \quad \frac{u_2}{K_2} = a \cdot \varepsilon \gg 1 \quad \rightarrow f_H = \frac{a \cdot \varepsilon \cdot a \cdot \varepsilon}{a\varepsilon+a \cdot \varepsilon} = \frac{a \cdot \varepsilon}{2} \quad (1.4)$$

The fold change (FC) or output dynamic range is defined as:

$$FC_{11/00} = \frac{f_H}{f_L} = \frac{\frac{a\cdot\varepsilon}{2}}{\frac{\varepsilon}{2}} = a \tag{2.1}$$

$$FC_{11/10} = \frac{f_H}{f_L} = \frac{\frac{a\cdot\varepsilon}{2}}{\frac{a}{a+1}\cdot\varepsilon} = \frac{a+1}{2} \tag{2.2}$$

$$FC = min\{FC_{11/00}, FC_{11/10}\} \;\Rightarrow FC = \frac{a+1}{2} \tag{2.3}$$

The equation that describes the AND logic gate based on protein-protein interaction is:

$$f(u_1, u_2) = \frac{\frac{u_1}{K_1}\cdot\frac{u_2}{K_2}}{1+\frac{u_1}{K_1}\cdot\frac{u_2}{K_2}} \tag{3}$$

The function *f* has two asymptotical values: f_L ➔ Low, and f_H ➔ High, which can be achieved when:

$$\frac{u_1}{K_1} \equiv \varepsilon \ll 1, \quad \frac{u_2}{K_2} = \varepsilon \ll 1 \quad \rightarrow f_L = \frac{\varepsilon^2}{1+\varepsilon^2} \tag{3.1}$$

$$\frac{u_1}{K_1} = a\cdot\varepsilon \gg 1, \; \frac{u_2}{K_2} = \varepsilon \ll 1 \quad \rightarrow f_L = \frac{a\cdot\varepsilon^2}{1+a\cdot\varepsilon^2} \tag{3.2}$$

$$\frac{u_1}{K_1} = \varepsilon \ll 1, \quad \frac{u_2}{K_2} = a\cdot\varepsilon \gg 1 \rightarrow f_L = \frac{a\cdot\varepsilon^2}{1+a\cdot\varepsilon^2} \tag{3.3}$$

$$\frac{u_1}{K_1} = a\cdot\varepsilon \gg 1, \; \frac{u_2}{K_2} = a\cdot\varepsilon \gg 1 \rightarrow f_H = \frac{a^2\cdot\varepsilon^2}{1+a^2\cdot\varepsilon^2} \tag{3.4}$$

The fold change (FC) or output dynamic range is defined as:

$$FC_{11/00} = \frac{f_H}{f_L} = \frac{\frac{a^2\cdot\varepsilon^2}{1+a^2\cdot\varepsilon^2}}{\frac{\varepsilon^2}{1+\varepsilon^2}} = \frac{a^2\cdot(1+\varepsilon^2)}{1+a^2\cdot\varepsilon^2} \tag{4.1}$$

$$FC_{11/10} = \frac{f_H}{f_L} = \frac{\frac{a^2\cdot\varepsilon^2}{1+a^2\cdot\varepsilon^2}}{\frac{a\cdot\varepsilon^2}{1+a\cdot\varepsilon^2}} = \frac{a\cdot(1+a\cdot\varepsilon^2)}{1+a^2\cdot\varepsilon^2} \tag{4.2}$$

$$FC = min\{FC_{11/00}, FC_{11/10}\} \;\Rightarrow FC = \frac{a\cdot(1+a\cdot\varepsilon^2)}{1+a^2\cdot\varepsilon^2} \tag{4.3}$$

The parameter “a” is almost equal to the input dynamic range (*IDR)*:

$$IDR \approx \frac{u_H}{u_L} = \frac{a\cdot\varepsilon}{\varepsilon} \;\Rightarrow IDR = a \tag{5}$$

We assumed that the two inputs have equal $IDRs$. Therefore, we can write the fold change for the two systems as:

AND logic gate based on MIN fuzzy lattice $FC_{MIN} = \frac{IDR+1}{2}$ (6.1)

AND logic gate based on protein-protein interaction $FC_{p-p} = \frac{IDR\cdot(1+IDR\cdot\varepsilon^2)}{1+IDR^2\cdot\varepsilon^2}$ (6.2)

Figure S4 shows the simulation results of Eq. 6.1 and Eq. 6.2. The simulation results indicate that the fold change of the AND logic gate based on the MIN fuzzy lattice is higher than that of the AND logic gate based on protein-protein interaction. Only for very low ε (*i.e.* u_i<<K_i), which is often challenging to implement in biological systems, the fold change of the AND logic gate based on protein-protein interaction is higher than that of the AND logic gate based on the MIN fuzzy lattice.

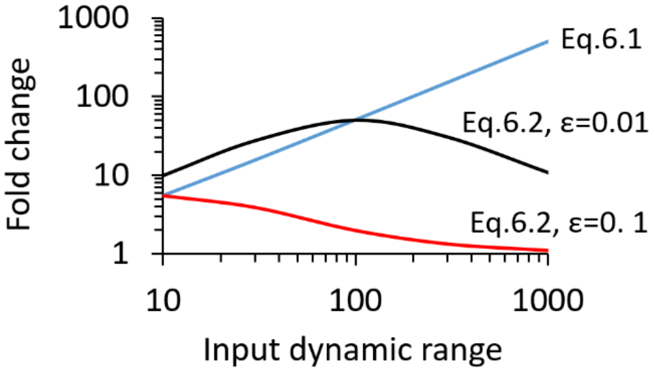

Supplementary Figure S4 Simulation results of fold change as a function of input dynamic range.

For further analysis, we compared the behavior of the developed AND logic gates with an ideal AND logic gate. The ideal AND logic gate is based on a smooth minimum function integrated with nonlinear activation functions for the inputs. Such an AND logic gate can be described as a hard minimum function, which performs logical operations ("if" loop) to a set of analog/digital numbers. While a smooth minimum function performs analog operation to a set of analog numbers (x_i). A smooth minimum function (https://en.wikipedia.org/wiki/Smooth_maximum) between two analog numbers can be expressed as:

$$f(u_1, u_2) = \frac{\frac{u_1}{K_1} \cdot e^{-\alpha \cdot u_1} + \frac{u_2}{K_2} \cdot e^{-\alpha \cdot u_2}}{e^{\alpha \cdot u_1} + e^{-\alpha \cdot u_2}}$$, where *α>>0*. (7)

To achieve a hard minimum function, we added a nonlinear activation function for every input (x_i):

$$u_i = \frac{x_i}{1+x_i}$$, where *i=1,2*. (8)

In our simulations we assumed that $K_1=K_2=K_i$. The simulation results show that the ideal AND gate (simulation results Fig. S5a-S5c) and the one developed and based on the MIN fuzzy lattice (simulation results Fig S5d- Fig S5) have similar behaviors. Both have a very weak dependency on the dissociation constant K_i. By contrast, the AND logic gate based on protein-protein interaction strongly depends on the K_i values (simulation results Fig S5g - Fig S5i). The parameter K_i can reduce the fold change and disturb the performance of the AND logic gate based on protein-protein interaction (Fig S6).

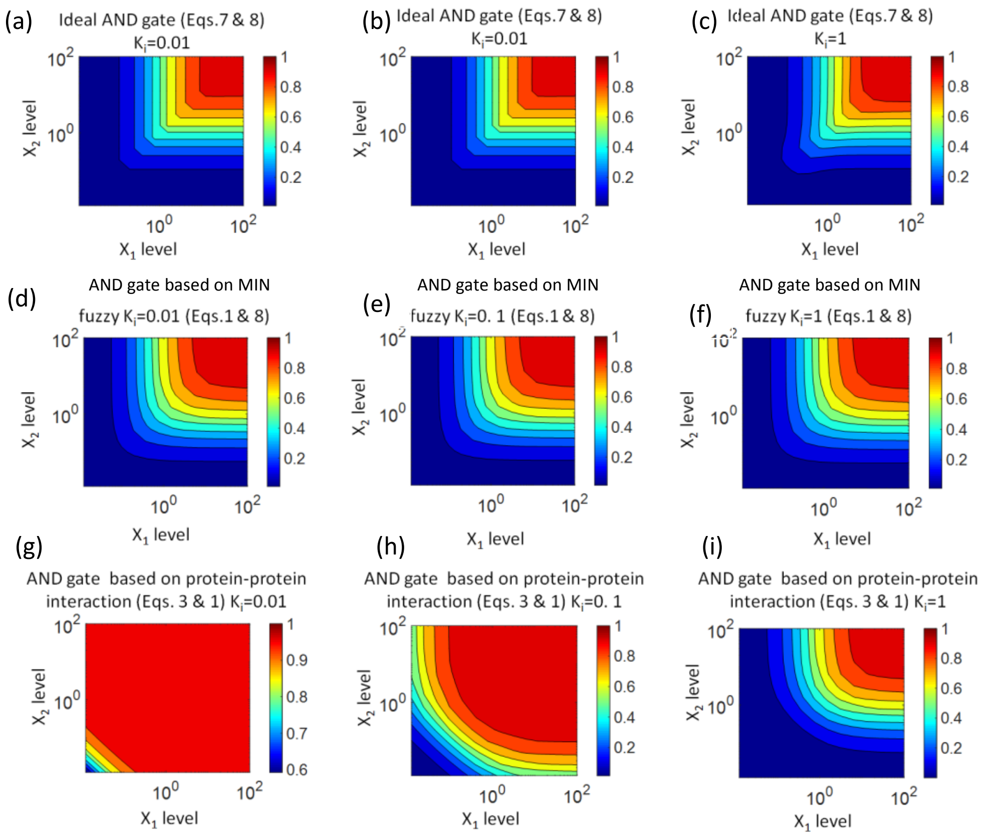

Supplementary Figure S5 Simulation results of AND logic gates

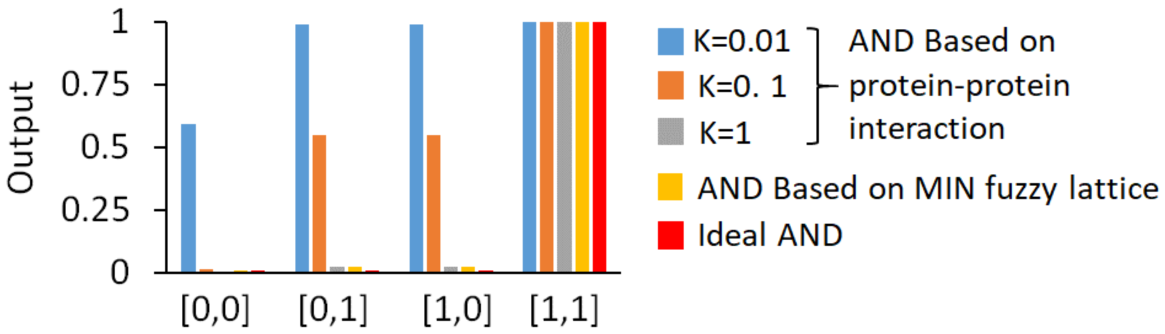

Supplementary Figure S6 Simulation results of AND logic gates represented as logic states

Section 5 - Programmable detection threshold circuit

Here we analyze how comparators with programmable detection thresholds can be combined together and used for biosensing applications. There are two types of circuits to report the values of detected input signals, namely analog and digital. In analog circuits, the reported value is proportional to the detected input. However, there are two main issues with using analog circuits for biosensing.

1. It is challenging to increase the input and output dynamic ranges of biological parts (1)
2. Analog signals are not reliable and any environmental changes can affect the results.

Digital circuits have only two computational levels (low and high), and therefore environmental changes will not strongly affect the results. However, the main disadvantage of the digital circuits is that the system cannot inform us about the detected input value. To solve this challenge, we suggest using sensors that behave in a digital manner with programmable detection thresholds (Fig. S7). The system includes two biosensors, each of which has a distinct AHL concentration level: 15 nM and 11,111 nM respectively. The system can detect various Arabinose levels (low, medium and high) based on the different detection thresholds of each biosensor (determined by AHL level) (Fig. 4a, 4c). For the two biosensors, we measured reference levels (no Arabinose is provided, I_{L0}, I_{H0}) and calculated the fold change (ON/OFF ratio, Fig. 4d).

For the *luxCDE-luxAB* spitting design (Fig. 3a), for each level of Arabinose (low, medium and high) we measured the signals of two biosensors and calculated the fold change (FC):

Arabinose= Low ➔ FC_L=1, FC_M=1, ➔ [0,0]
Arabinose= Medium ➔ FC_L=20, FC_H=1 ➔ [1,0]
Arabinose= High ➔ FC_L=20, FC_H=87 ➔ [1,1]

In a similar way for the circuit design by further splitting the *luxCDABE* cassette (Fig. 4b) we get:

Arabinose= Low ➔ FC_L=1, FC_M=1, ➔ [0,0]
Arabinose= Medium ➔ FC_L=18, FC_H=1 ➔ [1,0]
Arabinose= High ➔ FC_L=18, FC_H=35 ➔ [1,1]

The two circuits can be used to discretize continuous Arabinose concentration values into multiple distinct outputs, which allow us to report the analog value of the input using digital output bits.

The fold change of the second circuit has a weak dependency on AHL concentration and therefore it is more reliable and suitable for real world applications, as changes in AHL concentration can occur.

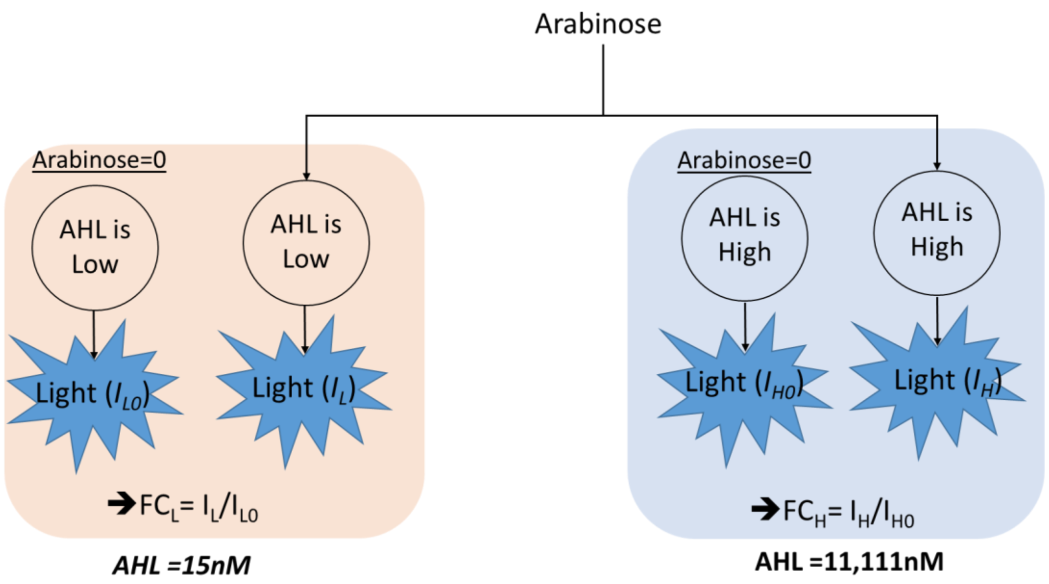

For the *luxCDE-luxAB* spitting design:

For further *luxA-luxC* splitting design:

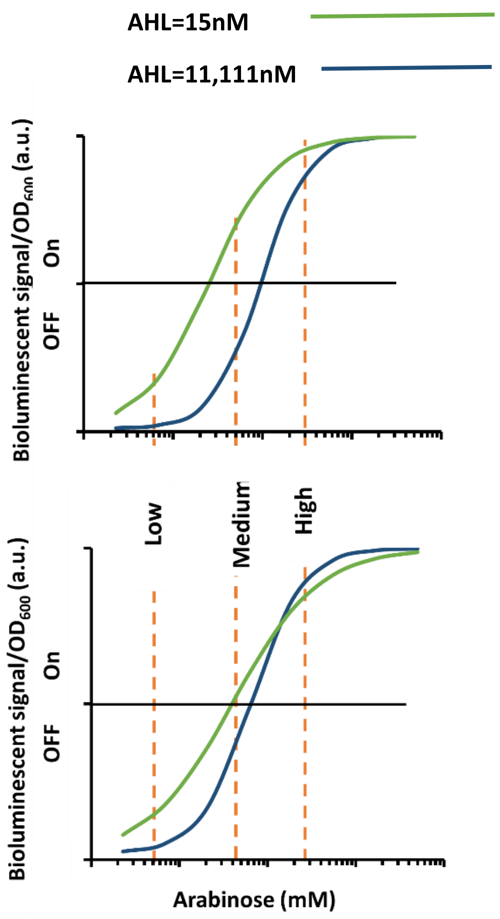

Supplementary Figure S7 Biosensors that behave in a digital manner with programmable detection thresholds

Section 6 - Further comparison of the *recA*-based bacterial biosensor and crosstalk-compensating circuit performance

From the data shown in Fig.5b of the main text we can conclude that the standard deviation of some experimental trials (σ_{max}) is larger than the first peak magnitude (H_1) (Fig.S8). For further comparison of the performance of the *recA*-based bacterial biosensor and crosstalk-compensating circuit, we ran a new experiment where the concentrations of NA and H_2O_2 were changed (conditions of first experiment: H_2O_2=25mg/l, NA= 2mg/l-4mg/l, conditions of second experiment: H_2O_2=50mg/l, NA=2mg/l-4mg/l) (Fig.S9). We calculated the magnitude of the first peak (H_1) and the second peak (H_2) for the two experiments (Table S8). From the experimental results we can see that in the circuit of P_{recA} alone the peak has a small magnitude and even disappeared in some cases. By contrast, the cross-talk compensating circuit permanently display two separated peaks.

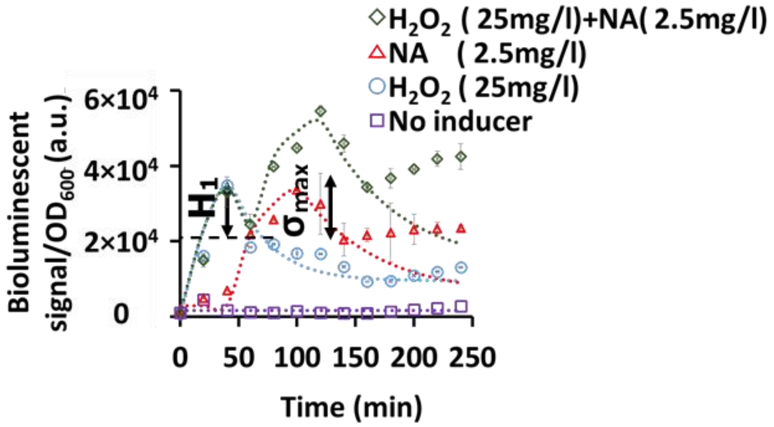

Supplementary Figure S8 Bioluminescent signal at time points following *recA* promoter induction includes H_2O_2 peak magnitude (H_1) and the maximal standard deviation (σ_{max}).

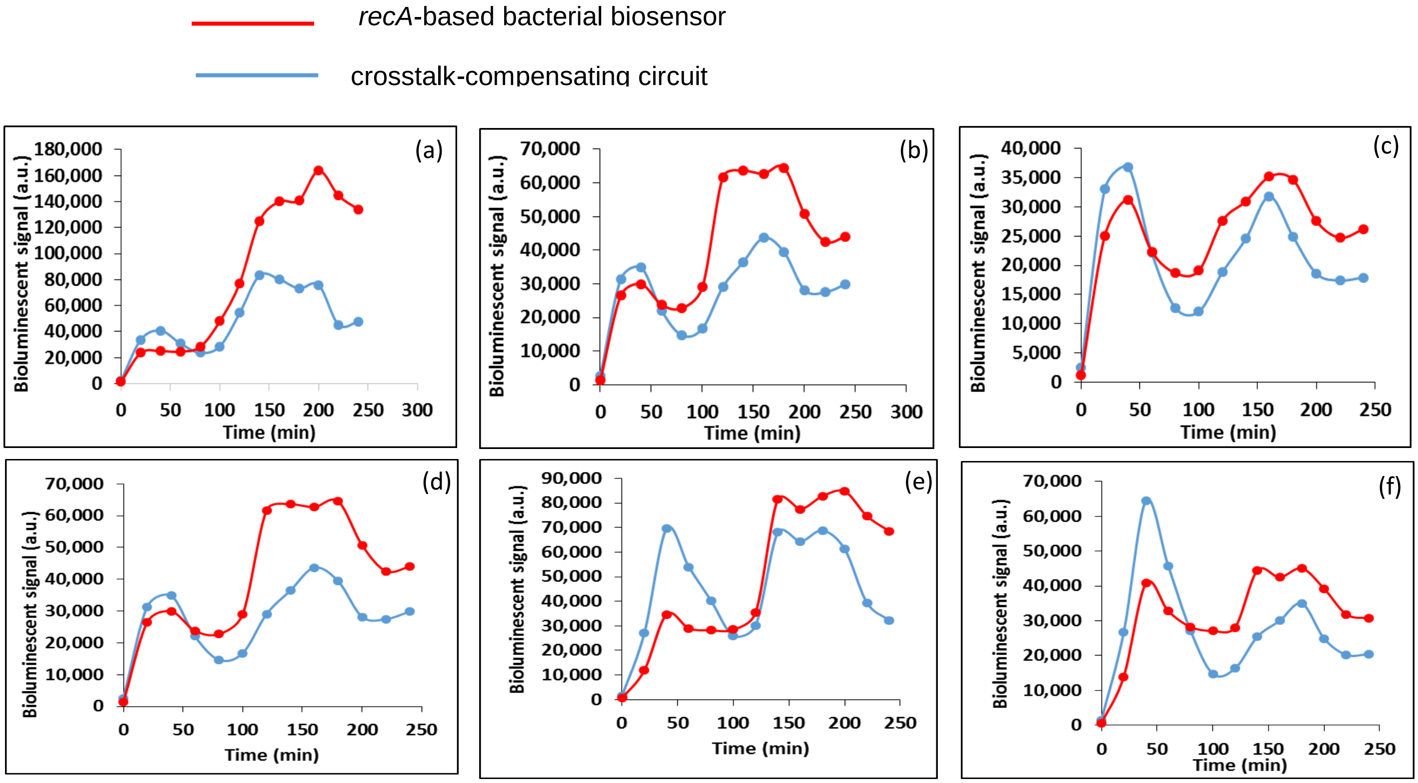

Supplementary Figure S9 The performance of the *recA*-based bacterial biosensor and crosstalk-compensating circuit for various H_2O_2 and NA concentrations: (a) H_2O_2 = 25mg/l, NA = 4mg/l; (b) H_2O_2 = 25mg/l, NA = 3mg/l; (c) H_2O_2 = 25mg/l, NA = 2mg/l; (d) H_2O_2 = 50mg/l, NA = 4mg/l; (e) H_2O_2 = 50mg/l, NA = 3mg/l; (f) H_2O_2 = 50mg/l, NA = 2mg/l.

Supplementary Table S10 The magnitude of peaks for various NA and H_2O_2 concentrations

**H_2O_2=25mg/l**	**NA=4mg/l**		**NA=3mg/l**		**NA=2mg/l**	
	**H_1 (%)**	**H_2 (%)**	**H_1 (%)**	**H_2 (%)**	**H_1 (%)**	**H_2 (%)**
**cross-talk compensating circuit**	72	251	137	197	204	163
***recA*-based bacterial biosensor**	-16	398	32	180	67	88
**H_2O_2=50mg/l**	**NA=4mg/l**		**NA=3mg/l**		**NA=2mg/l**	
	**H_1 (%)**	**H_2 (%)**	**H_1 (%)**	**H_2 (%)**	**H_1 (%)**	**H_2 (%)**
**cross-talk compensating circuit**	137	197	169	166	337	137
***recA*-based bacterial biosensor**	32	184	22	197	45	60

Section 7 Stochastic behavior of bacterial biosensors

There are three main noise sources in biosensors: (1) environmental noise resulting from random fluctuations in the physical conditions such as temperature, nutrients and cell density, (2) genetic noise resulting from random fluctuations in the levels of gene expression and (3) photo-detector noise resulting from the discrete nature of photons. Noise often disturbs the performance of bacterial biosensors by decreasing its resolution. That is, when a large noise is present, the minimum change in the input that the biosensor can detect is increased.

In this work, we modeled the impact of noise on the performance of bacterial biosensors.

We use the Langevin technique (2) to model the random fluctuations and the impact of the noise on the performance of bacterial biosensors. The Langevin equations can be systematically derived from the master equations in which random variables (signals) are treated as continuous variables, and stochastic effects are introduced by adding a time dependent term noise $\eta_j(t)$ to the deterministic dynamical equations. The term $\eta_j(t)$ describes the noise of each source, and is modeled as a Gaussian process (the fluctuations are uncorrelated in time) with the following statistics:

$$< \eta_j(t) >= 0$$
$$< \eta_j(t) \cdot \eta_j(t+\tau) >= \delta(\tau) \quad (1.1)$$

where $\delta(\tau)$ is the Dirac δ-function. For simplicity, we focus only on one type of noise source, in which it is assumed that the variance is equal to the mean signal as in a Poisson process. This statistical behavior can describe the intrinsic noise of gene expression (3) or the arrival of photons to photo-detectors (4). The Langevin equation of the measured bioluminescent signal ($I(t)$) for a bacterial biosensor can be described as:

$$\frac{dI}{dt} = \alpha - \frac{I}{\tau_{eff}} + \sqrt{2 \cdot q} \cdot \eta(t) \quad (1.2)$$

where τ_{eff} is the correlation time of the bioluminescent signal $I(t)$, and is equal to the τ_{eff} from Eq. 8 in the main text, q is the noise strength and is related to the diffusion constant, α is the rate of bioluminescent signal production. The variance of the signal is given by:

$$\overline{\Delta I^2} = \frac{2 \cdot q \cdot \tau_{eff}}{2} \quad (1.3)$$

The mean of the signal at the steady state is given by:

$$< I >= \alpha \cdot \tau_{eff} \quad (1.4)$$

We require that the mean is equal to the variance ($\overline{\Delta I^2} =< I >$), which in turn leads to $q = \alpha$. As it was explained in the main text (Fig. 5b), the bioluminescent signal of *recA* promoter is the summation of NA and H_2O_2 responses. The bioluminescent signal of *recA* by the NA is given by:

$$\frac{dI_{NA}}{d(t-\tau_{D1})} = \alpha - \frac{I_{NA}}{\tau_{eff}} \cdot exp\left(-\frac{t-\tau_{D2}}{\tau_1}\right) + \sqrt{2 \cdot \alpha} \cdot \eta(t - \tau_{D1}) \quad (1.5)$$

τ_{eff}*=25 min*: effective life time of *recA* promoter by NA
τ_1*=60 min*: consumption time of NA by bacteria
τ_{D1}*=60 min*: time delay for *recA* promoter activation by NA
τ_{D2}*=100 min*: time delay for the consumption of NA.
The values were extracted from experimental results (Fig. 5b).
α=10 protein/min, estimated according to the value of bioluminescent signal at steady state ~250 luciferase.

The bioluminescent signal of *recA* activation by H_2O_2 is given by:

$$\frac{dI_{H2O2}}{d(t-\tau_{D1})} = \alpha - \frac{I_{H2O2}}{\tau_{eff}} \cdot exp\left(-\frac{t-\tau_{D2}}{\tau_1}\right) + \sqrt{2\cdot\alpha}\cdot\eta(t-\tau_{D1}) \qquad (1.6)$$

τ_{eff}*=30 min*: effective life time of *recA* promoter by H_2O_2
τ_1*=30 min*: consumption time of H_2O_2 by bacteria
τ_{D1}*=0 min*: time delay for *recA* promoter activation by H_2O_2
τ_{D2}*=40 min*: time delay for the consumption of H_2O_2

The signal of *recA* activation by NA and H_2O_2 is given by:

$$I(t) = I_{NA}(t) + I_{H2O2}(t) \qquad (1.7)$$

The model of the crosstalk-compensating circuit (Fig. 5d-5g) is based on similar equations (Eq.1.5-1.7) and the same parameters, with a change of the time delay in Eq. 1.5 τ_{D1}*=100 min.* The stochastic simulation results recA promoter response to NA and H_2O_2 are shown in Fig. S10 (a) and (b), and the stochastic simulation results of crosstalk-compensating circuit are shown in Fig. S10 (c) and (d). The simulation results indicate that when noise is present, it is challenging to detect the first peak contributed by H_2O_2 using the *recA* promoter, by contrast to the crosstalk-compensating circuit.

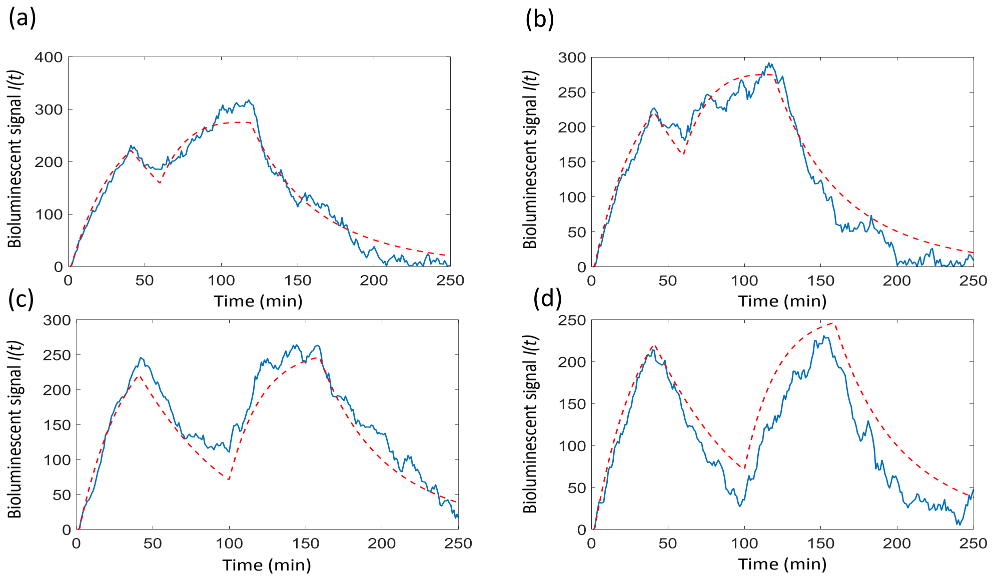

Supplementary Figure S10 Stochastic simulation of bacterial biosensors. (a) and (b) *recA* promoter (Fig. 5b), (c) and (d) crosstalk-compensating circuit (Fig. 5d). The red dots are the deterministic models. Simulation are based on Eqs. 1.5-1.7.

Section 8 Biological parts

Supplementary Table S11 List of biological parts

Part	DNA sequence
GFP	atgagtaaaggagaagaacttttcactggagttgtcccaattcttgttgaattagatggtgatgttaatgggcacaaattttct<br>gtcagtggagagggtgaaggtgatgcaacatacggaaaacttacccttaaatttatttgcactactggaaaactacctgtt<br>ccatggccaacacttgtcactactttcggttatggtgttcaatgctttgcgagatacccagatcatatgaaacagcatgacttt<br>ttcaagagtgccatgcccgaaggttatgtacaggaaagaactatatttttcaaagatgacgggaactacaagacacgtg<br>ctgaagtcaagtttgaaggtgatacccttgttaatagaatcgagttaaaaggtattgattttaaagaagatggaaacattctt<br>ggacacaaattggaatacaactataactcacacaatgtatacatcatggcagacaaacaaaagaatggaatcaaagtt<br>aacttcaaaattagacacaacattgaagatggaagcgttcaactagcagaccattatcaacaaaatactccaattggcg<br>atggccctgtccttttaccagacaaccattacctgtccacacaatctgccctttcgaaagatcccaacgaaaagagagac<br>cacatggtccttcttgagtttgtaacagctgctgggattacacatggcatggatgaactatacaaataa
LuxR	atgaaaaacataaatgccgacgacacatacagaataattaataaaattaaagcttgtagaagcaataatgatattaatc<br>aatgcttatctgatatgactaaaatggtacattgtgaatattatttactcgcgatcatttatcctcattctatggttaaatctgatatt<br>tcaatcctagataattaccctaaaaaatggaggcaatattatgatgacgctaatttaataaaatatgatcctatagtagattat<br>tctaactccaatcattcaccaattaattggaatatatttgaaaacaatgctgtaaataaaaaatctccaaatgtaattaaaga<br>agcgaaaacatcaggtcttatcactgggtttagtttccctattcatacggctaacaatggcttcggaatgcttagttttgcacat<br>tcagaaaaagacaactatatagatagtttatttttacatgcgtgtatgaacataccattaattgttccttctctagttgataattat<br>cgaaaaataaatatagcaaataataaatcaaacaacgatttaaccaaaagagaaaaagaatgtttagcgtgggcatg<br>cgaaggaaaaagctcttgggatatttcaaaaatattaggttgcagtgagcgtactgtcactttccatttaaccaatgcgcaa<br>atgaaactcaatacaacaaaccgctgccaaagtatttctaaagcaattttaacaggagcaattgattgcccatactttaaa<br>aattaataa
TetR	atgtccagattagataaaagtaaagtgattaacagcgcattagagctgcttaatgaggtcggaatcgaaggtttaacaac<br>ccgtaaactcgcccagaagctaggtgtagagcagcctacattgtattggcatgtaaaaaataagcgggctttgctcgacg<br>ccttagccattgagatgttagataggcaccatactcacttttgccctttagaaggggaaagctggcaagatttttacgtaata<br>acgctaaaagttttagatgtgctttactaagtcatcgcgatggagcaaaagtacatttaggtacacggcctacagaaaaa<br>cagtatgaaactctcgaaaatcaattagcctttttatgccaacaaggtttttcactagagaatgcattatatgcactcagcgct<br>gtggggcattttactttaggttgcgtattggaagatcaagagcatcaagtcgctaaagaagaaagggaaacacctactac<br>tgatagtatgccgccattattacgacaagctatcgaattatttgatcaccaaggtgcagagccagccttcttattcggccttg<br>aattgatcatatgcggattagaaaaacaacttaaatgtgaaagtgggtcctaa
P_{BAD}	aagaaaccaattgtccatattgcatcagacattgccgtcactgcgtcttttactggctcttctcgctaaccaaaccggtaacc<br>ccgcttattaaaagcattctgtaacaaagcgggaccaaagccatgacaaaaacgcgtaacaaaagtgtctataatcac<br>ggcagaaaagtccacattgattatttgcacggcgtcacactttgctatgccatagcatttttatccataagattagcggatcct<br>acctgacgctttttatcgcaactctctactgtttctccat
P_{lacO}	aattgtgagcggataacaattgacattgtgagcggataacaagatactgagcacatcagcaggacgcactgacc
P_{tetO}	Tccctatcagtgatagagattgacatccctatcagtgatagagatactgagcacatcagcaggacgcactgacc
Plux	acctgtaggatcgtacaggtttacgcaagaaaatggtttgttatagtcgaataaa
$Plux_{LBL}$	acctgtaggatcgtacaggtttacgcaagaaaatggtttgttactttcgaataaa
p15A	cggtcgttcgactgcggcgagcggaaatggcttacgaacggggcggagatttcctggaagatgccaggaagatactta<br>acagggaagtgagagggccgcggcaaagccgtttttccataggctccgcccccctgacaagcatcacgaaatctgac<br>gctcaaatcagtggtggcgaaacccgacaggactataaagataccaggcgtttccccctggcggctccctcgtgcgctct<br>cctgttcctgcctttcggtttaccggtgtcattccgctgttatggccgcgtttgtctcattccacgcctgacactcagttccgggta<br>ggcagttcgctccaagctggactgtatgcacgaaccccccgttcagtccgaccgctgcgccttatccggtaactatcgtctt<br>gagtccaacccggaaagacatgcaaaagcaccactggcagcagccactggtaattgatttagaggagttagtcttgaa

	gtcatgcgccggttaaggctaaactgaaaggacaagttttggtgactgcgctcctccaagccagttacctcggttcaaag<br>agttggtagctcagagaaccttcgaaaaaccgccctgcaaggcggttttttcgttttcagagcaagagattacgcgcaga<br>ccaaaacgatctcaagaagatcatcttattaatcagataaaatatttctagatttcagtgcaatttatctcttcaaatgtagcac<br>ctgaagtcagccccatacgatataagttgtt
pSC101	gtacgggttttgctgcccgcaaacgggctgttctggtgttgctagtttgttatcagaatcgcagatccggcttcaggtttgccg<br>gctgaaagcgctatttcttccagaattgccatgattttttccccacgggaggcgtcactggctcccgtgttgtcggcagctttg<br>attcgataagcagcatcgcctgtttcaggctgtctatgtgtgactgttgagctgtaacaagttgtctcaggtgttcaatttcatgtt<br>ctagttgctttgttttactggtttcacctgttctattaggtgttacatgctgttcatctgttacattgtcgatctgttcatggtgaacagc<br>tttaaatgcaccaaaaactcgtaaaagctctgatgtatctatcttttttacaccgttttcatctgtgcatatggacagttttccctttt<br>gatatctaacggtgaacagttgttctacttttgtttgttagtcttgatgcttcactgatagatacaagagccataagaacctcag<br>atccttccgtatttagccagtatgttctctagtgtggttcgttgtttttgcgtgagccatgagaacgaaccattgagatcatgctta<br>ctttgcatgtcactcaaaaattttgcctcaaaactggtgagctgaatttttgcagttaaagcatcgtgtagtgtttttcttagtccgt<br>tacgtaggtaggaatctgatgtaatggttgttggtattttgtcaccattcatttttatctggttgttctcaagttcggttacgagatcc<br>atttgtctatctagttcaacttggaaaatcaacgtatcagtcgggcggcctcgcttatcaaccaccaatttcatattgctgtaa<br>gtgtttaaatctttacttattggtttcaaaacccattggttaagccttttaaactcatggtagttattttcaagcattaacatgaactt<br>aaattcatcaaggctaatctctatatttgccttgtgagttttcttttgtgttagttcttttaataaccactcataaatcctcatagagta<br>tttgttttcaaaagacttaacatgttccagattatattttatgaatttttttaactggaaaagataaggcaatatctcttcactaaaa<br>actaattctaatttttcgcttgagaacttggcatagtttgtccactggaaaatctcaaagcctttaaccaaaggattcctgatttc<br>cacagttctcgtcatcagctctctggttgctttagctaatacaccataagcattttccctactgatgttcatcatctgagcgtattg<br>gttataagtgaacgataccgtccgttctttccttgtagggttttcaatcgtggggttgagtagtgccacacagcataaaattag<br>cttggtttcatgctccgttaagtcatagcgactaatcgctagttcatttgctttgaaaacaactaattcagacatacatctcaatt<br>ggtctaggtgattttaatcactataccaattgagatgggctagtcaatgataattactagtccttttcctttgagttgtgggtatct<br>gtaaattctgctagacctttgctggaaaacttgtaaattctgctagaccctctgtaaattccgctagacctttgtgtgttttttttgttt<br>atattcaagtggttataatttatagaataaagaaagaataaaaaaagataaaaagaatagatcccagccctgtgtataac<br>tcactactttagtcagttccgcagtattacaaaaggatgtcgcaaacgctgtttgctcctctacaaaacagaccttaaaacc<br>ctaaaggcttaagtagcaccctcgcaagctcgggcaaatcgctgaatattccttttgtctccgaccatcaggcacctgagt<br>cgctgtctttttcgtgacattcagttcgctgcgctcacggctctggcagtgaatgggggtaaatggcactacaggcgcctttta<br>tggattcatgcaaggaaactacccataatacaagaaaagcccgtcacgggcttctcagggcgttttatggcgggtctgct<br>atgtggtgctatctgactttttgctgttcagcagttcctgccctctgattttccagtctgaccacttcggattatcccgtgacaggt<br>cattcagactggctaatgcacccagtaaggcagcggtatcatcaacaggcttacccgtcttactgtccctagt
ColE1	cgttcggctgcggcgagcggtatcagctcactcaaaggcggtaatacggttatccacagaatcaggggataacgcagg<br>aaagaacatgtgagcaaaaggccagcaaaaggccaggaaccgtaaaaaggccgcgttgctggcgtttttccataggc<br>tccgcccccctgacgagcatcacaaaaatcgacgctcaagtcagaggtggcgaaacccgacaggactataaagata<br>ccaggcgtttccccctggaagctccctcgtgcgctctcctgttccgaccctgccgcttaccggatacctgtccgcctttctccc<br>ttcgggaagcgtggcgctttctcaatgctcacgctgtaggtatctcagttcggtgtaggtcgttcgctccaagctgggctgtgt<br>gcacgaaccccccgttcagcccgaccgctgcgccttatccggtaactatcgtcttgagtccaacccggtaagacacgact<br>tatcgccactggcagcagccactggtaacaggattagcagagcgaggtatgtaggcggtgctacagagttcttgaagtg<br>gtggcctaactacggctacactagaaggacagtatttggtatctgcgctctgctgaagccagttaccttcggaaaaagagt<br>tggtagctcttgatccggcaaacaaaccaccgctggtagcggtggtttttttgtttgcaagcagcagattacgcgcagaaa<br>aaaaggatctcaagaagatcctttgatcttttctacggggtctgacgctcagtggaacgaaaactcacgttaagggattttg<br>gtcatg
cmR	aaattacgccccgccctgccactcatcgcagtactgttgtaattcattaagcattctgccgacatggaagccatcacaaac<br>ggcatgatgaacctgaatcgccagcggcatcagcaccttgtcgccttgcgtataatatttgcccatggtgaaaacgggggg<br>cgaagaagttgtccatattggccacgtttaaatcaaaactggtgaaactcacccagggattggctgagacgaaaaacat<br>attctcaataaaccctttagggaaataggccaggttttcaccgtaacacgccacatcttgcgaatatatgtgtagaaactgc<br>cggaaatcgtcgtggtattcactccagagcgatgaaaacgtttcagtttgctcatggaaaacggtgtaacaagggtgaac<br>actatcccatatcaccagctcaccgtctttcattgccatacgaaattccggatgagcattcatcaggcgggcaagaatgtg<br>aataaaggccggataaaacttgtgcttatttttctttacggtctttaaaaaggccgtaatatccagctgaacggtctggttata<br>ggtacattgagcaactgactgaaatgcctcaaaatgttctttacgatgccattgggatatatcaacggtggtatatccagtg

	atttttttctccattttagcttccttagctcctgaaaatctcgataactcaaaaaatacgcccggtagtgatcttatttcattatggt<br>gaaagttggaacctcttacgtgcccgatcaa
ampR	gtaaacttggtctgacagttaccaatgcttaatcagtgaggcacctatctcagcgatctgtctatttcgttcatccatagttgcct<br>gactccccgtcgtgtagataactacgatacgggagggcttaccatctggccccagtgctgcaatgataccgcgagaccc<br>acgctcaccggctccagatttatcagcaataaaccagccagccggaagggccgagcgcagaagtggtcctgcaacttt<br>atccgcctccatccagtctattaattgttgccgggaagctagagtaagtagttcgccagttaatagtttgcgcaacgttgttgc<br>cattgctacaggcatcgtggtgtcacgctcgtcgtttggtatggcttcattcagctccggttcccaacgatcaaggcgagtta<br>catgatcccccatgttgtgcaaaaaagcggttagctccttcggtcctccgatcgttgtcagaagtaagttggccgcagtgtta<br>tcactcatggttatggcagcactgcataattctcttactgtcatgccatccgtaagatgcttttctgtgactggtgagtactcaac<br>caagtcattctgagaatagtgtatgcggcgaccgagttgctcttgcccggcgtcaatacgggataataccgcgccacata<br>gcagaactttaaaagtgctcatcattggaaaacgttcttcggggcgaaaactctcaaggatcttaccgctgttgagatcca<br>gttcgatgtaacccactcgtgcacccaactgatcttcagcatcttttactttcaccagcgtttctgggtgagcaaaaacagga<br>aggcaaaatgccgcaaaaaagggaataagggcgacacggaaatgttgaatactcatactcttcctttttcaatattattga<br>agcatttatcagggttattgtctcatgagcggatacatatttgaatgtatttagaaaaataaacaaataggggttccgcgcac<br>atttccccgaaaagtgccacct
kanR	tcgaaccccagagtcccgctcagaagaactcgtcaagaaggcgatagaaggcgatgcgctgcgaatcgggagcggc<br>gataccgtaaagcacgaggaagcggtcagcccattcgccgccaagctcttcagcaatatcacgggtagccaacgctat<br>gtcctgatagcggtccgccacacccagccggccacagtcgatgaatccagaaaagcggccattttccaccatgatattc<br>ggcaagcaggcatcgccatgggtcacgacgagatcctcgccgtcgggcatgcgcgccttgagcctggcgaacagttc<br>ggctggcgcgagcccctgatgctcttcgtccagatcatcctgatcgacaagaccggcttccatccgagtacgtgctcgctc<br>gatgcgatgtttcgcttggtggtcgaatgggcaggtagccggatcaagcgtatgcagccgccgcattgcatcagccatga<br>tggatactttctcggcaggagcaaggtgagatgacaggagatcctgccccggcacttcgcccaatagcagccagtccct<br>tcccgcttcagtgacaacgtcgagcacagctgcgcaaggaacgcccgtcgtggccagccacgatagccgcgctgcct<br>cgtcctgcagttcattcagggcaccggacaggtcggtcttgacaaaaagaaccgggcgcccctgcgctgacagccgg<br>aacacggcggcatcagagcagccgattgtctgttgtgcccagtcatagccgaatagcctctccacccaagcggccgga<br>gaacctgcgtgcaatccatcttgttcaatcatgcgaaacgatcctcatcctgtctcttgatcagatcttgatcccctgcgccat<br>cagatccttggcggcaagaaagccatccagtttactttgcagggcttcccaaccttaccagagggcgccccagctggca<br>attcc
AraC	atggctgaagcgcaaaatgatcccctgctgccgggatactcgtttaacgcccatctggtggcgggtttaacgccgattgag<br>gccaacggttatctcgatttttttatcgaccgaccgctgggaatgaaaggttatattctcaatctcaccattcgcggtcaggg<br>ggtggtgaaaaatcagggacgagaatttgtctgccgaccgggtgatattttgctgttcccgccaggagagattcatcacta<br>cggtcgtcatccggaggctcgcgaatggtatcaccagtgggtttactttcgtccgcgcgcctactggcatgaatggcttaac<br>tggccgtcaatatttgccaatacgggtttctttcgcccggatgaagcgcaccagccgcatttcagcgacctgtttgggcaaa<br>tcattaacgccgggcaaggggaagggcgctattcggagctgctggcgataaatctgcttgagcaattgttactgcggcgc<br>atggaagcgattaacgagtcgctccatccaccgatggataatcgggtacgcgaggcttgtcagtacatcagcgatcacct<br>ggcagacagcaattttgatatcgccagcgtcgcacagcatgtttgcttgtcgccgtcgcgtctgtcacatcttttccgccagc<br>agttagggattagcgtcttaagctggcgcgaggaccaacgcattagtcaggcgaagctgcttttgagcactacccggatg<br>cctatcgccaccgtcggtcgcaatgttggttttgacgatcaactctatttctcgcgagtatttaaaaaatgcaccggggccag<br>cccgagcgagtttcgtgccggttgtgaagaaaaagtgaatgatgtagccgtcaagttgtcataa
LuxI	atgactataatgataaaaaaaatcggatttttggcaattccatcggaggagtataaaggtattctaagtcttcgttatcaagtg<br>tttaagcaaagacttgagtgggacttagttgtagaaaataaccttgaatcagatgagtatgataactcaaatgcagaatat<br>atttatgcttgtgatgatactgaaaatgtaagtggatgctggcgtttattacctacaacaggtgattatatgctgaaaagtgtttt<br>tcctgaattgcttggtcaacagagtgctcccaaagatcctaatatagtcgaattaagtcgttttgctgtaggtaaaaatagctc<br>aaagataaataactctgctagtgaaattacaatgaaactatttgaagctatatataaacacgctgttagtcaaggtattaca<br>gaatatgtaacagtaacatcaacagcaatagagcgatttttaaagcgtattaaagttccttgtcatcgtattggagacaaag<br>aaattcatgtattaggtgatactaaatcggttgtattgtctatgcctattaatgaacagtttaaaaaagcagtcttaaatgctgc<br>aaacgacgaaaactacgctttagtagcttaataactctgatagtgctagtgtagatctc

rrnB T1 terminator	caaataaaacgaaaggctcagtcgaaagactgggcctttcgttttatctgttgtttgtcggtgaacgctctcctgagtaggac<br>aaat
PkatG	cgaaatgagggcgggaaaataaggttatcagccttgttttctccctcattacttgaaggatatgaagctaaaaccctttttat<br>aaagcatttgtccgaattcggacataatcaaaaaagcttaattaagatcaatttgatctacatctctttaaccaacaatatgt<br>aagatctcaactatcgcatccgtggattaattcaattataacttctctctaacgctgtgtatcgtaacggtaacactgtagagg<br>ggagcacattgatgagcacgtcagacgatatccataacaccacagccactggcaaatgcccgttccatcagggcggtc<br>acgaccagagtgcgggggcgggcacaaccactcgcgactggtggccaaatcaacttcgtgttgacctgttaaaccaac<br>attctaatcgttctaacccactgggtgaggactttgactaccgcaaagaattcagcaaattagattactacggcctgaaaa<br>aagatctgaaagccctgttgacagaatctcaaccgtggtggccagccgactggggcagttacgccggtctgtttattcgta<br>tggcctggcacggcgcggggacttaccgttcaatcgatggacgcggtggcgcgggtcgtggtcagcaacgtt
PrecA	agagaagcctgtcggcaccgtctggtttgcttttgccactgcccgcggtgaaggcattacccggcgggatgcttcagcgg<br>cgaccgtgatgcggtgcgtcgtcaggctactgcgtatgcattgcagaccttgtggcaacaatttctacaaaacacttgatac<br>tgtatgagcatacagtataattgcttcaacagaacatattgactatccggtattacccggcatgacaggagtaaaaatggc<br>tatcgacgaaaacaaacagaaagcg
LuxC	atgactaaaaaaatttcattcattattaacggccaggttgaaatctttcccgaaagtgatgatttagtgcaatccattaattttg<br>gtgataatagtgtttacctgccaatattgaatgactctcatgtaaaaaacattattgattgtaatggaaataacgaattacggt<br>tgcataacattgtcaattttctctatacggtagggcaaagatggaaaaatgaagaatactcaagacgcaggacatacatt<br>cgtgacttaaaaaaatatatgggatattcagaagaaatggctaagctagaggccaattggatatctatgattttatgttctaa<br>aggcggcctttatgatgttgtagaaaatgaacttggttctcgccatatcatggatgaatggctacctcaggatgaaagttatg<br>ttcgggcttttccgaaaggtaaatctgtacatctgttggcaggtaatgttccattatctgggatcatgtctatattacgcgcaattt<br>taactaagaatcagtgtattataaaaacatcgtcaaccgatccttttaccgctaatgcattagcgttaagttttattgatgtaga<br>ccctaatcatccgataacgcgctctttatctgttatatattggccccaccaaggtgatacatcactcgcaaaagaaattatgc<br>gacatgcggatgttattgtcgcttggggagggccagatgcgattaattgggcggtagagcatgcgccatcttatgctgatgt<br>gattaaatttggttctaaaaagagtctttgcattatcgataatcctgttgatttgacgtccgcagcgacaggtgcggctcatga<br>tgtttgtttttacgatcagcgagcttgtttttctgcccaaaacatatattacatgggaaatcattatgaggaatttaagttagcgtt<br>gatagaaaaacttaatctatatgcgcatatattaccgaatgccaaaaaagattttgatgaaaaggcggcctattctttagttc<br>aaaaagaaagcttgtttgctggattaaaagtagaggtggatattcatcaacgttggatgattattgagtcaaatgcaggtgt<br>ggaatttaatcaaccacttggcagatgtgtgtaccttcatcacgtcgataatattgagcaaatattgccttatgttcaaaaaa<br>ataagacgcaaaccatatctatttttccttgggagtcatcatttaaatatcgagatgcgttagcattaaaaggtgcggaaag<br>gattgtagaagcaggaatgaataacatatttcgagttggtggatctcatgacggaatgagaccgttgcaacgattagtgac<br>atatatttctcatgaaaggccatctaactatacggctaaggatgttgcggttgaaatagaacagactcgattcctggaaga<br>agataagttccttgtatttgtcccataa
LuxD	atggaaaatgaatcaaaatataaaaccatcgaccacgttatttgtgttgaaggaaataaaaaaattcatgtttgggaaac<br>gctgccagaagaaaacagcccaaagagaaagaatgccattattattgcgtctggttttgcccgcaggatggatcattttgc<br>tggtctggcggaatatttatcgcggaatggatttcatgtgatccgctatgattcgcttcaccacgttggattgagttcagggac<br>aattgatgaatttacaatgtctataggaaagcagagcttgttagcagtggttgattggttaactacacgaaaaataaataac<br>ttcggtatgttggcttcaagcttatctgcgcggatagcttatgcaagcctatctgaaatcaatgcttcgtttttaatcaccgcagt<br>cggtgttgttaacttaagatattctcttgaaagagctttagggtttgattatctcagtctacccattaatgaattgccggataatct<br>agattttgaaggccataaattgggtgctgaagtctttgcgagagattgtcttgattttggttgggaagatttagcttctacaatta<br>ataacatgatgtatcttgatataccgtttattgcttttactgcaaataacgataattgggtcaagcaagatgaagttatcacatt<br>gttatcaaatattcgtagtaatcgatgcaagatatattctttgttaggaagttcgcatgacttgagtgaaaatttagtggtcctgc<br>gcaatttttatcaatcggttacgaaagccgctatcgcgatggataatgatcatctggatattgatgttgatattactgaaccgt<br>catttgaacatttaactattgcgacagtcaatgaacgccgaatgagaattgagattgaaaatcaagcaatttctctgtcttaa
LuxA	atgaaatttggaaactttttgcttacataccaacctccccaattttctcaaacagaggtaatgaaacgtttggttaaattaggtc<br>gcatctctgaggagtgtggttttgataccgtatggttactggagcatcatttcacggagtttggtttgcttggtaacccttatgtc<br>gctgctgcatatttacttggcgcgactaaaaaattgaatgtaggaactgccgctattgttcttcccacagcccatccagtacg<br>ccaacttgaagatgtgaatttattggatcaaatgtcaaaaggacgatttcggtttggtatttgccgagggctttacaacaagg<br>actttcgcgtattcggcacagatatgaataacagtcgcgccttagcggaatgctggtacgggctgataaagaatggcatg

	acagagggatatatggaagctgataatgaacatatcaagttccataaggtaaaagtaaaccccgcggcgtatagcaga<br>ggtggcgcaccggtttatgtggtggctgaatcagcttcgacgactgagtgggctgctcaatttggcctaccgatgatattaa<br>gttggattataaatactaacgaaaagaaagcacaacttgagctttataatgaagtggctcaagaatatgggcacgatattc<br>ataatatcgaccattgcttatcatatataacatctgtagatcatgactcaattaaagcgaaagagatttgccggaaatttctg<br>gggcattggtatgattcttatgtgaatgctacgactatttttgatgattcagaccaaacaagaggttatgatttcaataaaggg<br>cagtggcgtgactttgtattaaaaggacataaagatactaatcgccgtattgattacagttacgaaatcaatcccgtggga<br>acgccgcaggaatgtattgacataattcaaaaagacattgatgctacaggaatatcaaatatttgttgtggatttgaagcta<br>atggaacagtagacgaaattattgcttccatgaagctcttccagtctgatgtcatgccatttcttaaagaaaaacaacgttcg<br>ctattatattag
LuxB	atgaaatttggattgttcttccttaacttcatcaattcaacaactgttcaagaacaaagtatagttcgcatgcaggaaataac<br>ggagtatgttgataagttgaattttgaacagattttagtgtatgaaaatcatttttcagataatggtgttgtcggcgctcctctga<br>ctgtttctggttttctgctcggtttaacagagaaaattaaaattggttcattaaatcacatcattacaactcatcatcctgtcgcc<br>atagcggaggaagcttgcttattggatcagttaagtgaagggagatttattttagggtttagtgattgcgaaaaaaaagatg<br>aaatgcatttttttaatcgcccggttgaatatcaacagcaactatttgaagagtgttatgaaatcattaacgatgctttaacaa<br>caggctattgtaatccagataacgatttttatagcttccctaaaatatctgtaaatccccatgcttatacgccaggcggacctc<br>ggaaatatgtaacagcaaccagtcatcatattgttgagtgggcggccaaaaaaggtattcctctcatctttaagtgggatg<br>attctaatgatgttagatatgaatatgctgaaagatataaagccgttgcggataaatatgacgttgacctatcagagataga<br>ccatcagttaatgatattagttaactataacgaagatagtaataaagctaaacaagagacgcgtgcatttattagtgattat<br>gttcttgaaatgcaccctaatgaaaatttcgaaaataaacttgaagaaataattgcagaaaacgctgtcggaaattatacg<br>gagtgtataactgcggctaagttggcaattgaaaagtgtggtgcgaaaagtgtattgctgtcctttgaaccaatgaatgattt<br>gatgagccaaaaaaatgtaatcaatattgttgatgataatattaagaagtaccacatggaatatacctaa
LuxE	atgacttcatatgttgataaacaagaaattacagcaagctcagaaattgatgatttgatttttcgagcgatccattagtgtggt<br>cttacgacgagcaggaaaaaatcagaaagaaacttgtgcttgatgcatttcgtaatcattataaacattgtcgagaatatc<br>gtcactactgtcaggcacacaaagtagatgacaatattacggaaattgatgacatacctgtattcccaacatcggtttttaa<br>gtttactcgcttattaacttctcaggaaaacgagattgaaagttggtttaccagtagcggcacgaatggtttaaaaagtcag<br>gtggcgcgtgacagattaagtattgagagactcttaggctctgtgagttatggcatgaaatatgttggtagttggtttgatcat<br>caaatagaattagtcaatttgggaccagatagatttaatgctcataatatttggtttaaatatgttatgagtttggtggaattgtta<br>tatcctacgacatttaccgtaacagaagaacgaatagattttgttaaaacattgaatagtcttgaacgaataaaaaatcaa<br>gggaaagatctttgtcttattggttcgccatactttatttatttactctgccattatatgaaagataaaaaaatctcattttctggag<br>ataaaagcctttatatcataaccggaggcggctggaaaagttacgaaaaagaatctctgaaacgtgatgatttcaatcat<br>cttttatttgatactttcaatctcagtgatattagtcagatccgagatatatttaatcaagttgaactcaacacttgtttctttgagg<br>atgaaatgcagcgtaaacatgttccgccgtgggtatatgcgcgagcgcttgatcctgaaacgttgaaacctgtacctgatg<br>gaacgccggggttgatgagttatatggatgcgtcagcaaccagttatccagcatttattgttaccgatgatgtcgggataatt<br>agcagagaatatggtaagtatcccggcgtgctcgttgaaattttacgtcgcgtcaatacgaggacgcagaaagggtgtg<br>ctttaagcttaaccgaagcgtttgatagttga

Supplementary References

1. Daniel,R., Rubens,J.R., Sarpeshkar,R. and Lu,T.K. (2013) Synthetic analog computation in living cells. *Nature*, **497**, 619–623.
2. Gardiner,C.W. (1997) Handbook of stochastic methods for physics, chemistry, and the natural sciences Springer, Berlin.
3. Ozbudak,E.M., Thattai,M., Kurtser,I., Grossman,A.D. and van Oudenaarden,A. (2002) Regulation of noise in the expression of a single gene. *Nat. Genet.*, **31**, 69–73.
4. Van der Ziel,A. (1976) Noise in measurements J. Wiley & Sons Inc. UMI, Michigan.
